# The Path Towards Effective Long-Lasting Tissue-Targeted Prime/Pull/Keep Herpes Simplex Therapeutic Vaccines

**DOI:** 10.3390/vaccines13090908

**Published:** 2025-08-27

**Authors:** Afshana Quadiri, Yassir Lekbach, Elhoucine Elfatimi, Swayam Prakash, Hawa Vahed, Sweta Karan, Azizur Rehman, Sarah Xue Le Ng, Chhaya Maurya, Reilly Chow, Lbachir BenMohamed

**Affiliations:** 1 Laboratory of Cellular and Molecular Immunology, Gavin Herbert Eye Institute, School of Medicine, University of California, Irvine, CA 92697, USA; aquadiri@hs.uci.edu (A.Q.); ylekbach@hs.uci.edu (Y.L.); eelfatim@hs.uci.edu (E.E.); fswayamp@hs.uci.edu (S.P.); hvahed@uci.edu (H.V.); skaran1@hs.uci.edu (S.K.); azizurr@uci.edu (A.R.); ngsx@uci.edu (S.X.L.N.); cmaurya@uci.edu (C.M.); reillyac@uci.edu (R.C.); 2 Institute for Immunology, School of Medicine, University of California, Irvine, CA 92697, USA; 3 Department of Vaccines and Immunotherapies, TechImmune, LLC, University Lab Partners, Irvine, CA 92660, USA; 4 Ophthalmology Research Laboratories, Irvine, CA 92697, USA

**Keywords:** PPK vaccines, herpes simplex virus (HSV-1 and HSV-2), clinical trials, vaccines, immunotherapeutic, symptomatic, asymptomatic, epitopes

## Abstract

The development of vaccines against many infectious diseases has been a great success of medical science over the last century. However, despite numerous efforts, effective vaccines for herpes simplex virus types 1 and 2 (HSV-1 and HSV-2) remain elusive. Since 1920s, a range of therapeutic vaccine candidates, primarily focusing on neutralizing antibodies, have failed to confer robust and durable protective immunity against recurrent herpes. Recent advances in omics, artificial intelligence, and deep learning have opened new horizons for the rational design of tissue-targeted herpes vaccine strategies for inducing potent and durable HSV-specific CD4^+^ and CD8^+^ T_RM_ cell immunity at both the sensory ganglia (central immunity), the site of latency/reactivation cycle, and the mucocutaneous epithelial tissues (peripheral immunity), the site of viral replication that causes herpetic lesions. Prime/Pull/Keep ocular and genital herpes vaccine candidates (PPK vaccines) have recently shown success in pre-clinical animal model trials of recurrent ocular and genital herpes. These PPK vaccines used “asymptomatic” epitopes/antigens to prime CD4^+^ and CD8^+^ T cells (Prime); primed T cells are then pulled towards the infected central and peripheral epithelial tissues using T cell-attracting chemokines, such as CXCL11 (Pull), followed by survival cytokines (IL-2, IL-7 and/or IL-15) or mucosal chemokines (CXCL17 and/or CCL28) to maintain the “pulled” tissue-resident T cells longer within infected tissues (Keep). We discuss recent efforts in designing a clinically adapted, all-in-one PPK mucosal therapeutic vaccine that would require a single administration to sequentially trigger all three PPK steps of priming, recruiting, and maintaining antiviral, tissue-resident, protective T cells at the primary sites of viral entry and latency.

## 1. Introduction

**Figure 1:**
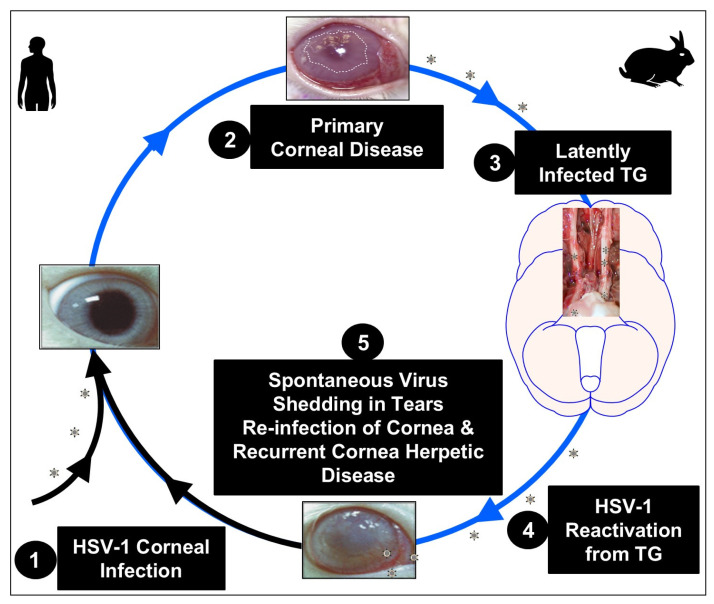
Ocular HSV-1 life cycle. HSV-1 is transmitted by ocular contact and causes an acute primary herpetic disease that is quickly resolved by the immune system. However, few viral particles travel via the axons of sensory neurons to the trigeminal ganglia, where they establish latency in the sensory neurons. Spontaneous reactivation of HSV-1 from latently infected TG triggers shedding of the virus in tears and re-infection of the cornea, causing recurrent ocular herpetic disease. HSV-1 re-infection and replication in corneal epithelium causes mild to potentially blinding ocular herpetic disease. Asterisk (*) symbolizes the movement of the HSV-1 virus through different phases of infection.

Herpes simplex virus types 1 (HSV-1) and 2 (HSV-2) are common and lifelong infections [1]. HSV-1 primarily causes oral infections, while HSV-2 is mainly responsible for genital manifestations [1]. Both HSV-1 and HSV-2 are highly prevalent globally, with a significant impact on public health due to their potential for recurrent outbreaks and, in some instances, complications such as neonatal herpes in newborns when transmitted during childbirth [1]. An estimated 3.8 billion people under the age of 50 (64%) globally have HSV-1, and 520 million people aged 15–49 (13%) have HSV-2 infection [1]. An estimated 205 million people aged 15–49 (5.3%) experienced at least one symptomatic episode of genital herpes in 2020 [2]. HSV-1 is primarily transmitted through oral contact and is the leading cause of orolabial herpes, also known as cold sores, as well as primary ocular infection and recurrent ocular herpes keratitis (Figure 1). The virus is contagious during periods of active viral replication, typically marked by visible lesions [3]. However, asymptomatic shedding from the oral mucosa occurs on approximately 5–10% of days, even in the absence of visible lesions, presenting a risk of transmission despite the lack of symptoms [4]. The frequency and severity of recurrent herpes simplex labialis outbreaks can vary significantly between individuals [4]. Recurrent ocular herpetic disease is a leading infectious cause of corneal blindness in developed nations, resulting from reactivation of latent HSV-1 from sensory neurons in the trigeminal ganglia (TG), followed by anterograde transportation back to the cornea via nerve termini, leading to shedding in tears, and ultimately causing potentially blinding recurrent corneal herpetic disease (Figure 1). No immunotherapeutic is currently available [5]. Symptomatic patients must rely on sustained antiviral drugs (i.e., Acyclovir and derivatives) and undergo corneal transplantation in instances of severe scarring [4]. Over the last 25 years, only a single subunit vaccine strategy, adjuvanted recombinant HSV glycoproteins gB and gD, has been tested in clinical trials [6]. This parenterally injected protein/adjuvant vaccine failed to meet the primary endpoint of reducing recurrent herpes disease, despite inducing strong systemic HSV-specific CD4^+^ and CD8^+^ T cell responses [7].

**Figure 2:**
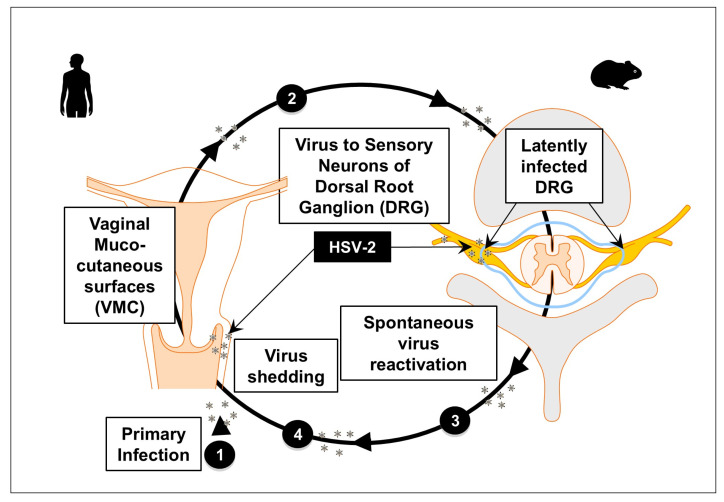
Genital HSV-2 life cycle. The HSV-2 life cycle is similar in human and guinea pig hosts. After primary vaginal infection, HSV-2 enters sensory nerves innervating the skin or mucosa and undergoes a retrograde axonal transport to the sensory neurons of dorsal root ganglia where it establishes a lifelong latent infection. During sporadic and spontaneous reactivations, HSV-2 travels back via anterograde axonal transport towards the peripheral epidermis to shed asymptomatically in the genital tract or to cause symptomatic recurrent genital herpetic disease. Asterisk (*) symbolizes the movement of the HSV-1 virus through different phases of infection.

**Figure 3:**
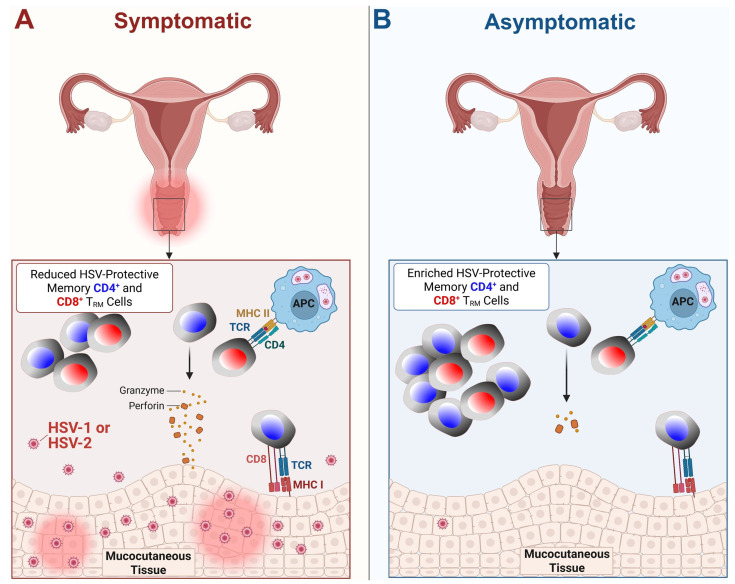
Schematic representation of the mucosal immune landscape in (**A**) symptomatic versus (**B**) asymptomatic genital herpes patients. The mucosa of asymptomatic individuals is characterized by an abundance of HSV cross-protective memory CD4^+^ and CD8^+^ T_RM_ cells that provide robust local immune surveillance, effectively suppress HSV-2 reactivation, and prevent symptomatic disease (right panel). In contrast, the left panel depicts the mucosal sites of symptomatic patients, which harbor fewer HSV cross-reactive T_RM_ cells that fail to control HSV replication, resulting in poor viral control and contributing to tissue damage and recurrent disease.

Genital herpes simplex virus infection may also be asymptomatic or manifest as a painful genital ulcerative disease that can produce multiple effector molecules and cytokines, which enhance their ability to control the virus (Figure 2 and Figure 3). Symptoms include localized pain, itching, dysuria, and the appearance of vesicular or ulcerative lesions in the genital or anal region [8]. Recurrent episodes may occur, accompanied by systemic symptoms such as fever, malaise, and lymphadenopathy during primary outbreaks [9]. Both HSV-1 and HSV-2 can cause severe disease in immunocompromised individuals [10]. Moreover, recent studies indicate that up to 10% of genital herpes cases are now attributable to HSV-1, underscoring the importance of including HSV-1 in genital herpes diagnostics [10]. The psychological and social impact of recurrent genital herpes is considerable; in fact, HSV-2 infection increases the risk of acquiring and transmitting HIV infection [11]. Primary infection typically occurs at mucocutaneous surfaces, followed by replication in epithelial cells (Figure 2). When the initial infection heals, the virus spreads to sensory nerve cells through the retrograde axonal transport of the virus to the corresponding sensory ganglia, where it remains dormant until reactivation occurs [12]. The virus establishes latency in neuronal ganglia, from which it can periodically reactivate, leading to recurrent disease and ongoing transmission [13].

Virus shedding and re-infection of the VMC tissues may be either (1) asymptomatic (ASYMP) with mild or unrecognized lesions [14]; or (2) symptomatic (SYMP) with severe and painful mucocutaneous genital lesions leading to complications including urinary retention and substantial psychological illness [15] (Figure 2). Despite widely used methodologies to control genital herpes, its spread remains an epidemic in some populations. It is commonly believed that the widespread use of an effective vaccine can prevent or reduce symptomatic disease and eliminate or at least limit asymptomatic viral shedding, which may, in turn, help control recurrent genital herpes disease [16]. However, despite several efforts, a safe and effective genital herpes vaccine remains unavailable.

There is currently no practical way to proactively prevent initial infection due to the lack of a licensed vaccine. The existing treatments, which include antiviral medications, can only manage outbreaks and reduce the transmission risk to a limited extent, failing to eliminate the latent virus within the body, which makes long-term prevention difficult [17]. The ability of HSV to establish latency within nerve cells makes it challenging to eradicate the virus, even with complete antiviral treatment [18]. Therefore, the development of anti-herpes medications has had little apparent impact on the epidemiology of herpes. At the same time, developing effective vaccines against herpes has been highly challenging, mainly because HSV-1 and HSV-2 have complex life cycles, and infection can remain clinically dormant in the body for extended periods [19]. Moreover, HSV employs multiple strategies to evade host immunity, including downregulation of MHC molecules, inhibition of interferon signaling, and modulation of apoptosis [20]. These features complicate the development of effective vaccines, as both humoral and cellular immune responses are required for protection.

Over the past 25 years, efforts to develop a herpes simplex subunit vaccine have explored multiple antigens, various delivery systems, and adjuvants, yet without success. Relatively few subunit vaccine strategies have advanced to human clinical trials, with the most notable involving subunit vaccines targeting HSV glycoproteins B and D (gB and gD) [21]. The Herpevac Trial for Women, a significant Phase III study evaluating a gD2-based vaccine, demonstrated partial efficacy against genital infection caused by HSV-1, but failed to protect against HSV-2 [21]. These failures underscore the need for herpes simplex subunit vaccine experiments to move beyond just trying antigens, delivery systems, adjuvants, and routes of systemic/parenteral administrations and instead explore innovative tissue-targeted vaccine strategies that would induce or boost local T cell immunity at the mucocutaneous tissues (peripheral immunity) and the ganglia, the sites of HSV latency and reactivation (central immunity) [22].

A critical barrier remains the limited understanding of the precise immune responses required for durable protection and viral control. Understanding how HSV evades the immune system is crucial, as studying the complex interactions between the virus and the host can help guide the development of vaccines that trigger strong protective immune responses. Developing effective vaccines or strategies largely depends on identifying the key immune correlates, which will guide the design of candidates that elicit the right quality and magnitude of immune responses, as well as strategies that recruit these immune cells to combat viral latency, immune evasion, and reactivation.

## 2. Immune Responses to Herpes Simplex Virus

The immune responses against HSV are complex and multifactorial [23,24]. Understanding these immune responses is crucial for guiding the rational design of effective HSV vaccines and therapeutic strategies [23,25]. Evidence generated from human studies and animal models of herpesvirus infection has demonstrated a critical role for both innate and adaptive immunity in controlling primary and latent infections [26,27]. Innate immune responses, including the activity of natural killer (NK) cells, macrophages, and the production of type I interferons—primarily IFN-α and IFN-β—play a critical role in controlling viral replication and limiting disease severity [28]. The integrity of mucosal barriers and the presence of local immune effectors influence the susceptibility to infection and disease severity [8,29]. The mucosal barriers and antimicrobial peptides attempt to block viral entry, while pattern recognition receptors detect viral components, inducing the production of type I interferons and pro-inflammatory cytokines [30,31]. These pro-inflammatory molecules function to recruit other inflammatory cells into infected tissues and activate antigen-presenting cells to induce adaptive immunity [32,33,34]. HSV has evolved mechanisms to counteract the interferon response, which include the expression of viral proteins such as ICP0, ICP27, US11 VHS, ICP47, and ICP34.5 that hamper interferon signaling and the expression of ISGs [35,36,37]. NK cells and dendritic cells (DCs) play crucial roles in priming adaptive immunity [38]. HSV can impair DC function by interfering with antigen presentation and inducing DC apoptosis [39]. Despite this, DCs contribute to the antiviral cytokine milieu by secreting type I interferons and IL-12 [40,41]. Tissue-resident macrophages also recognize HSV and contribute to early containment through phagocytosis and the secretion of cytokines [42]. These innate responses help shape and direct the adaptive immune system, including the activation of antigen-specific B and T cells [43].

The humoral arm of the adaptive immune response primarily contributes to protection through the production of neutralizing antibodies, which typically target surface glycoproteins that limit viral spread at mucocutaneous surfaces and block re-infection. CD4^+^ and CD8^+^ T cells control and destroy virally infected cells [44,45]. CD8^+^ T cells can directly kill infected cells and suppress viral reactivation within the sensory ganglia. In contrast, CD4^+^ T cells support both B cell maturation and enhance CD8^+^ T cell responses, helping to orchestrate the local immune environment. Animal models have demonstrated that effector CD8^+^ T cells surround HSV-1-infected ganglia and control latency; they also surround nerve termini in HSV-2-infected genital epithelium, complementing the function of CD4^+^ T cells in viral clearance from genital lesions [46]. An increased number of HSV-specific CD8^+^ T_RM_ cells, expressing high levels of tissue homing and tissue residency receptors (i.e., CXCR3, IL-2R/IL-15R, CD69, and CD103), that reside in the TG of HSV-1-infected HLA-A*0201 transgenic rabbits, were associated with decreased virus reactivation in the TG and reduced virus shedding in the cornea [47]. Individuals who are HSV seronegative elicited peripheral HSV-specific T cell responses despite the absence of infection [48]. This suggests that virus-specific T cells in these individuals have been stimulated by exposure and provide protection [48]. Zhu et al. identified a distinct subset of tissue-resident effector memory CD8αα^+^ T cells that remain at the dermal-epidermal junction after the classical CD8αβ^+^ T cells have declined, acting as sentinel cells that mediate viral clearance upon reactivation [49].

**Figure 4:**
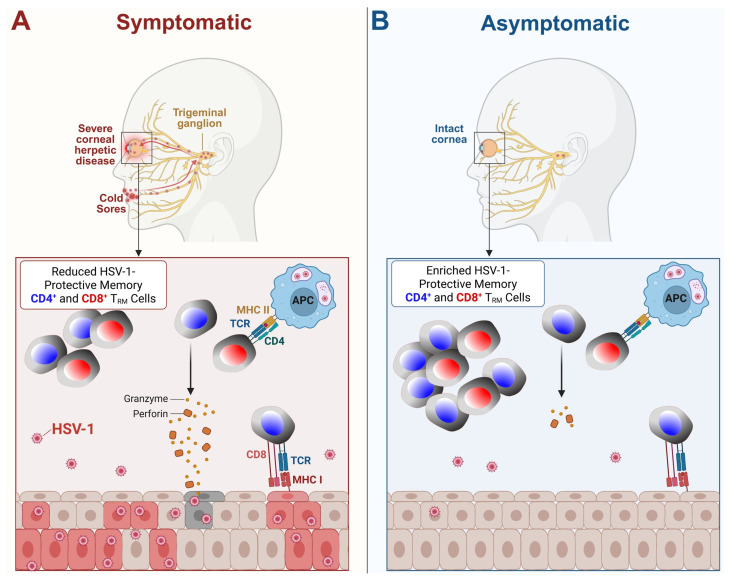
Schematic representation of the mucosal immune landscape in (**A**) symptomatic versus (**B**) asymptomatic ocular herpes patients. The mucosa of asymptomatic individuals is characterized by an abundance of HSV cross-protective memory CD4^+^ and CD8^+^ T_RM_ cells that provide robust local immune surveillance, effectively suppress HSV-1 reactivation, and prevent symptomatic disease (right panel). In contrast, the left panel depicts the mucosal sites of symptomatic patients, which harbor fewer HSV cross-reactive T_RM_ cells that fail to control HSV replication, resulting in poor viral control and contributing to tissue damage and recurrent disease.

Furthermore, people who frequently experience symptoms of HSV tend to have a weaker ability to produce IFN-γ in response to the virus [20]. Alternatively, individuals who produce higher levels of IFN-γ after an outbreak tend to experience the subsequent recurrence later [20]. Such individuals maintain a higher proportion of differentiated, polyfunctional T cells that can produce multiple effector molecules and cytokines, which enhance their ability to control the virus (Figure 3 and Figure 4). Asymptomatic individuals tend to have more T cells with distinct functional profiles and epitope specificities compared to symptomatic individuals, suggesting that antigens that preferentially activate T cells in asymptomatic HSV carriers may play a crucial role in controlling the disease [17]. In contrast, symptomatic individuals tend to have more undifferentiated and dysfunctional HSV-specific CD8^+^ T cells, which may exhibit characteristics of cellular senescence and exhaustion [50]. These immune dynamics play a crucial role in both systemic and mucosal HSV infections (Figure 3 and Figure 4).

Neutrophilic granulocytes constitute a significant immune cell population in the normal murine cornea [51]. In contrast, populations of T cells expressing either αβ or γδ T cell receptors (TCRs) have been detected in the vaginal epithelium [52]. HSV-specific CD4^+^ and CD8^+^ T cells are activated in the iliac lymph nodes following genital HSV-2 inoculation [52]. They can be detected later in the genital epithelium at a time coincident with virus clearance. While CD4^+^ T cells are the predominant lymphocyte subpopulation responsible for HSV-2 clearance from the genital epithelium, virus clearance can be mediated by other cell types in the absence of CD4^+^ T cells [53]. Although γδ T cells have been shown to exhibit lytic activity and secrete IFN-γ, the role of this cell population in the clearance of HSV-2 and the protection of the genital epithelium is unclear [52].

Despite these responses, HSV evades immunity by downregulating MHC molecules, inhibiting antigen presentation and interferon signaling, and establishing latency in sensory neurons [52]. During latency, viral gene expression is minimal, allowing multiple viral evasion mechanisms that have contributed to their evolutionary success [19]. In addition, most vaccines have provided limited protection against herpes, due to their induction of primarily antibody responses and the absence of a robust T cell component in their formulations. These challenges have informed past HSV vaccine strategies, which have aimed to reduce symptomatic disease, viral shedding, and recurrence.

## 3. Animal Models for Pre-Clinical Testing of Therapeutic Herpes Vaccine Candidates

Understanding the role of tissue-resident CD4^+^ and CD8^+^ T_RM_ cells in reducing herpes simplex reactivation requires an animal model that accurately reflects spontaneous virus reactivation, virus shedding, and recurrent herpes ocular and genital disease as they occur in humans [54]. Animal models offer the opportunity to study the phenotype, function, transcriptome, and specificity of immune responses induced by therapeutic vaccines longitudinally while also offering critical insights into the mechanisms of protection they confer [55,56]. A significant hindrance to developing a therapeutic vaccine for herpes has been the selection of a suitable animal model [55,56]. Selecting an appropriate animal model is crucial for vaccine development, as the model should closely replicate the characteristics of human HSV infection to enable a meaningful evaluation of vaccine-induced protection and durability.

### 3.1. Non-Human Primate (NHP) Models

NHPs closely resemble humans in anatomical, physiological, and immunological aspects, making them valuable for studying HSV pathogenesis and evaluating vaccine responses [57]. Despite their high cost and ethical constraints, their genetic similarity to humans enables the modeling of key disease features that are difficult to replicate in small animals. Old World NHPs such as the rhesus macaque have been used to model both HSV-1 and HSV-2 infections [57]. However, oral infection with virulent HSV-1 in NHPs resulted in low-level viral replication and elicited only modest immune responses, highlighting the challenges of modeling HSV-1 in these systems [58]. Ming Lo et al. developed a rhesus macaque model of intravaginal HSV-2 infection that mimics subclinical infection in women, demonstrating features of acute infection, persistence, spontaneous reactivation, and local mucosal inflammation, which make it a valuable tool for studying vaccine-induced immune control [49]. A study used Cebus apella, a New World primate, to model genital HSV-2 infection [59]. These animals displayed vesicular lesions and T cell responses, and, notably, HSV-2 infection increased susceptibility to vaginal HIV infection, creating a unique co-infection model [59]. Although several studies have tested vaccines and antiviral therapies in NHPs using intravaginal challenge models, the limited number of animals involved often restricts conclusions.

### 3.2. Mouse Model for Pre-Clinical Testing of Therapeutic Herpes Vaccine Candidates

The commonly used animal models for evaluation of HSV-2 candidate vaccines are mice and guinea pigs. The mouse model offers several advantages, including genetic tractability, availability of immunological tools, and the ability to use transgenic or knockout strains to dissect immune mechanisms in detail [60]. Mice are treated with progesterone, followed by an intravaginal HSV challenge, and successful infection is demonstrated through the development of local lesions and viral replication [60]. The model allows detailed characterization of CD4^+^ and CD8^+^ T cell responses, antigen-specific recall responses, and the role of cytokines such as IFN-γ and IL-15 in shaping mucosal immunity [60]. Studies in SV129 mice and SV129 mice lacking the type I interferon receptor have demonstrated the critical role of IFN-α/β signaling in antiviral defense against HSV-2 [61]. Moreover, murine models have been instrumental in identifying tissue-resident memory T cells in the vaginal mucosa following HSV-2 infection or vaccination, which makes mice a foundational pre-clinical platform for screening HSV vaccine candidates and investigating immune correlates of protection [61].

#### UV-B-Induced Recurrent Ocular Herpes in HLA Transgenic Mouse Model for Pre-Clinical Testing of Therapeutic Ocular Herpes Vaccine Candidates

Understanding the role of TG-resident CD4^+^ and CD8^+^ T_RM_ cells in reducing virus reactivation requires an animal model that accurately reflects the virus reactivation, virus shedding in tears, and recurrent ocular herpes as it occurs in humans [62]. While HSV-1 can infect mouse TG, the virus does not spontaneously reactivate in this model, unlike in humans. A previously established “humanized” HLA double-transgenic mouse model enables virus reactivation, virus shedding in tears, and recurrent ocular herpes following a 60 s exposure to UV-B light. The HLA Tg mouse expresses human HLA class I and class II instead of the mouse MHC class I and class II [63] and develops human-like CD4^+^ and CD8^+^ T cell responses to HLA-A*0201 restricted epitopes [64,65,66,67,68,69,70]. This unique “humanized” mouse model enables the testing of the protective efficacy of vaccine candidates bearing HLA-restricted human CD4^+^ and CD8^+^ T_RM_ cell epitopes in controlling UV-B-induced HSV-1 reactivation, as measured ex vivo by virus shedding in tears and in vitro by quantifying reduced virus reactivation in mouse TG explants. In conclusion, single and repetitive UV-B-induced reactivation in HLA double Tg mice constitutes a unique small animal model for studying HSV-1 reactivation and the role of TG-resident CD4^+^ and CD8^+^ T_RM_ cells in controlling recurrent ocular herpes.

### 3.3. Rabbit Model of Spontaneous Recurrent Ocular Herpes for Pre-Clinical Testing of Therapeutic Ocular Herpes Vaccine Candidates

Rabbits exhibit a close resemblance to humans in terms of HSV-1 spontaneous reactivation, disease progression, and immune response, making them a relevant animal model for studying ocular HSV-1 infection [71,72,73,74]. HSV-1 shedding in rabbits occurs both spontaneously and in response to local or systemic stimuli, mirroring the ∼35% spontaneous shedding rate observed in humans [63,75]. Most HSV-1 strains induce acute ocular infection, latency in TG, and spontaneous shedding in rabbits. This model is widely used due to (i) similarities between rabbit and human ocular mucosal immunity; (ii) comparable T cell-mediated ocular diseases, including herpetic conjunctivitis and stromal keratitis; (iii) rabbit conjunctival-associated lymphoid tissue (CALT) closely resembling human CALT, unlike mice; (iv) the large rabbit cornea and conjunctiva, which facilitate mucosal immunity studies; and (v) the growing availability of rabbit-specific immunological reagents, enabling detailed analysis of T cell responses. Several methods are being employed to induce herpetic corneal lesions in rabbits. The large eyes of rabbits produce abundant tears, providing sufficient tissue for viral and immunological analysis [76]. New Zealand. White and Dutch Belted rabbits are commonly used in ocular herpes studies. For vision-related research, rabbits with non-pigmented eyes are preferred. However, compared to mice, rabbits remain expensive and challenging to breed.

#### HLA Transgenic Rabbit Model of Spontaneous Recurrent Ocular Herpes for Pre-Clinical Testing of Therapeutic Ocular Herpes Vaccine Candidates

While ocular HSV-1 infection in mice led to TG infection, the virus does not spontaneously reactivate from the TG to re-infect the cornea in the mouse models (as reviewed in [77]). Recurrent ocular herpes does not occur spontaneously in mice, unlike in rabbits. Consequently, the “humanized” HLA Tg rabbit model, which develops spontaneous virus reactivation, virus shedding in tears, and recurrent ocular herpes, as occurs in humans, may be the preferred animal model for studying therapeutic herpes vaccines against ocular herpes [76]. We have been utilizing the rabbit model for the last 20 years and demonstrated its ability in testing the therapeutic efficacy of candidate vaccines against recurrent ocular herpes shedding [72,76]. While viral shedding into tears occurs frequently in rabbits, recurrent disease is infrequent (~10%) [72,76]. Moreover, the HLA Tg rabbit expresses human HLA class I instead of the rabbit MHC class I [63,72,76,78] and develops human-like CD8^+^ T cell responses to HLA-A*0201-restricted epitopes [63,72,76,79,80]. Although state-of-the-art rabbit immunology still lags behind that of mouse models, over the past decade, numerous reagents have been identified that have been instrumental in characterizing the phenotype, transcriptome, and function of rabbit CD8^+^ T_EM_, T_RM,_ and T_CM_ cells [63,72,76,80,81,82,83]. These reagents allow for a unique opportunity to characterize the frequency and function of these T cell subsets in the rabbit model [72,76]. Previously, we demonstrated that human HLA-A*02:01 tetramers can be readily used to accurately quantify the frequency of HLA-A*02:01-restricted epitope-specific CD8^+^ T_RM_ cells in HLA Tg rabbits [63,72,73,78,80]. Another advantage of the HLA Tg rabbit model is that it enables kinetic studies to track TG-resident T_RM_ cell frequencies and function/exhaustion in therapeutically vaccinated rabbits. However, similar to humans, recurrent ocular herpetic disease is rare in rabbits. Hence, the determination of recurrent corneal disease in vaccinated HLA-Tg rabbits following vaccination cannot be done in rabbits, as that would require hundreds of animals [80]. Instead, we are currently studying the effect of the PPK therapeutic vaccine on controlling spontaneous HSV-1 reactivation, as measured ex vivo by virus shedding in tears and in vitro by quantifying reduced virus reactivation in TG explants of vaccinated HLA-Tg rabbits. Stopping or reducing measurable virus shedding in the tears would be a strong indication of an efficacious therapeutic PPK vaccine against recurrent ocular herpes disease. In conclusion, the HLA-Tg rabbit offers a unique small animal model for investigating the role of TG-resident CD8^+^ T_RM_ cells in controlling recurrent ocular herpes and evaluating therapeutic vaccine candidates.

### 3.4. Guinea Pig Model of Genital Herpes as a Small Animal Model for Pre-Clinical Testing of Therapeutic Genital Herpes Vaccine Candidates

Due to the ethical and practical limitations in obtaining genital tract (GT) and dorsal root ganglia (DRG) samples and biopsies from HSV-2-infected patients, animal models offer the opportunity to study longitudinally the phenotype, function, transcriptome, and specificity of both GT- and DRG-resident T cells induced by therapeutic vaccine candidates [27]. However, a significant hindrance to developing a therapeutic vaccine for genital herpes has been the selection of a suitable animal model. Consensus exists that the guinea pig model that develops spontaneous virus reactivation, virus shedding in the GT, and human-like recurrent genital herpes disease is the gold standard and the most suitable small animal model for pre-clinical testing of therapeutic vaccine candidates that are translatable to humans [8,27,84,85,86,87,88,89,90,91,92,93]. Unlike guinea pigs, HSV-2 latently infected mice do not develop spontaneous recurrent genital herpes [66,69,94,95,96]. The guinea pigs do not require pre-treatment with medroxyprogesterone (also known as Depo-Provera) ahead of vaginal infection [55]. The lack of synchronization of their estrus cycle mimics conditions in humans [55]. Unlike guinea pigs, mice often require treatment with Depo-Provera to synchronize them into a diestrus state before being infected with HSV-2. The guinea pig model helps in investigating the role of immune responses and assessing the efficacy of vaccines targeting genital herpes.

#### Unprecedented Phenotypic, Functional, and Transcriptional B and T Cell Assays Are Now Possible in the Guinea Pig Model

A significant deficit of the guinea pig model is the lack of immunological reagents to evaluate cellular immune responses. For over three decades, it has not been technically feasible to perform phenotypic, functional, and transcriptional profiling of memory CD4^+^ and CD8^+^ T cell subsets in the guinea pig model, due to the unavailability of monoclonal antibodies (mAbs) specific to guinea pigs’ T cell markers, cytokines, and chemokines. Over the last five years, we have pushed the boundaries of T cell immunology in the guinea pig model [84,92]. Our laboratory has developed cutting-edge assays for phenotypic and functional characterization of circulating and tissue-resident CD4^+^ and CD8^+^ T cells in the guinea pig model [8,84,85,86,87,88,89,90,91,92]. We have now developed a panel of new mAbs for the CyToF assay together with the single-cell scRNA-Seq assay specific to guinea pig effector, regulatory, and memory CD4^+^ and CD8^+^ T cells, thereby allowing unprecedented opportunities to assess the phenotype and function of CD4^+^ and CD8^+^ T cells within the DRG and GT of HSV-2-infected and vaccinated guinea pigs [84,92]. Functional T cell assays, including IFN-γ-ELISpot, CFSE-based proliferation, surface markers of T cell activation (CD25, CD44, CD69, and CRTAM), and T cell exhaustion (PD-1, LAG-3, PSGL-1, and TIM-3), and intracellular cytokines can now all be assessed in the guinea pig model [84,92]. This allows unprecedented opportunities to determine the phenotype and function of CD4^+^ and CD8^+^ T cells within the DRG and GT of HSV-2-infected and vaccinated guinea pigs [84,92].

### 3.5. Tree Shrew Models

The tree shrew (Tupaia belangeri), a small mammal in the Tupaiidae family, is susceptible to many human viral pathogens. Genomic analysis studies suggest that tree shrews are more closely related to primates than to rodents [97]. Juvenile tree shrews are susceptible to HSV, exhibiting hepatitis-like symptoms and high HSV titers in the liver and spleen [98]. More recently, Li and colleagues demonstrated that tree shrews inoculated with HSV-1 exhibited encephalitis symptoms, further confirming the susceptibility of tree shrews to HSV-1 [60,65].

## 4. Herpes Simplex Virus Vaccine Strategies

**Figure 5:**
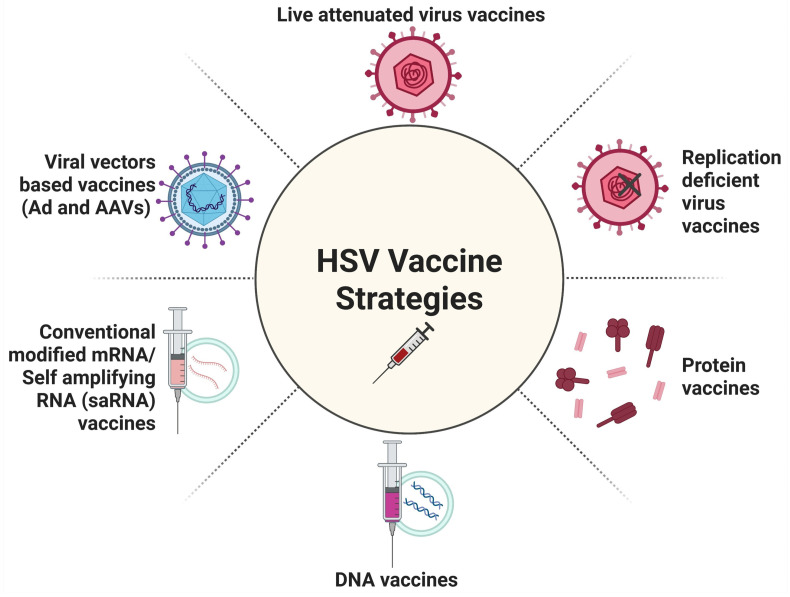
Illustration of the HSV vaccine strategies. The figure summarizes the herpes simplex virus vaccine strategies that have been used or explored so far, both in prophylactic and therapeutic settings, across pre-clinical and clinical studies.

Virus shedding and reactivation may be either (i) asymptomatic with mild or unrecognized lesions, or (ii) symptomatic with severe and painful lesions [4]. Although widely used methodologies to control herpes, including antiviral drugs (such as Acyclovir and its derivatives), education, and other measures, herpes infection remains an epidemic in some populations [4]. It is commonly believed that the widespread use of an effective vaccine can prevent or reduce symptomatic disease and eliminate or at least limit asymptomatic viral shedding, which may in turn help control the herpes simplex epidemic [4]. However, despite numerous efforts, a safe and effective herpes vaccine remains unavailable. Recent advances in understanding effective anti-herpes immune responses have led to the development of multiple novel vaccine approaches [84]. These strategies can be broadly categorized into prophylactic (preventive) and therapeutic (treatment-oriented) approaches. In this section, we discuss the progress of different vaccine strategies, recent developments in the pre-clinical pursuit of a safe and effective herpes simplex vaccine, and review subunit/peptide, vectored/DNA/RNA, and live-attenuated vaccine technologies (Table 1 and Figure 5). Several academic laboratories and commercial entities are currently working on developing a safe and effective herpes simplex vaccine in pre-clinical animal models and human trials.

### 4.1. Live-Attenuated but Replication Competent Vaccines

The development of a safe and effective herpes simplex vaccine, along with other vaccine platforms, involves the use of attenuated or modified viruses that can elicit robust immune responses. Live-attenuated vaccines have been the most effective vaccines in combating human and animal viral infections throughout medical history [99]. The repertoire of these successes includes the eradication of smallpox, poliomyelitis, measles, mumps, rubella, and rotavirus. A live-attenuated varicella-zoster virus vaccine is widely used worldwide and is highly efficacious in controlling viral reactivation [99]. The live varicella vaccine is safe and well-tolerated. The success of the VZV live-attenuated vaccines provides a primary example suggesting that a similar approach may be efficacious in combating herpes simplex infections, which, like VZV, establish latency in neurons.

**Figure 6:**
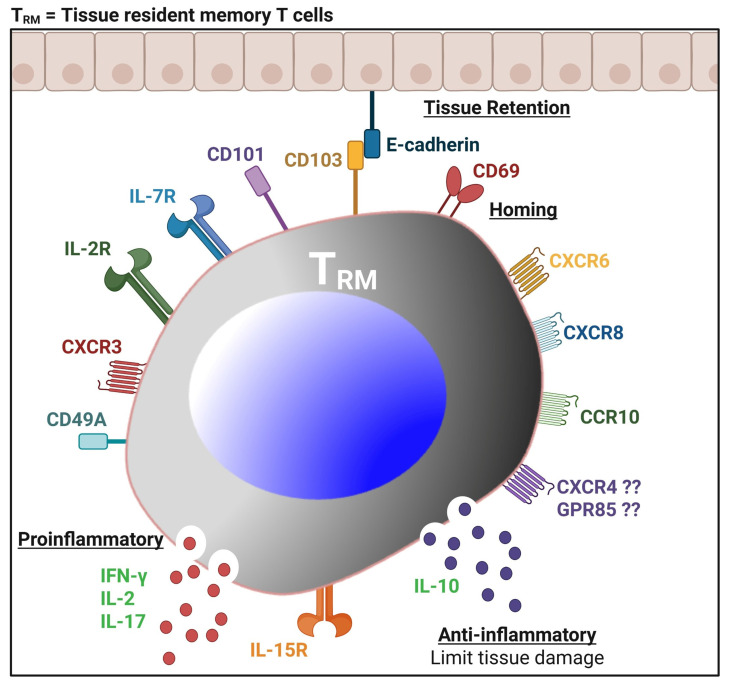
Phenotypic and functional attributes of tissue-resident memory T (T_RM_) cells in HSV infection. Tissue-resident memory CD4^+^ and CD8^+^ T_RM_ cells are lodged within peripheral tissues as sentinels, including the mucosal lining and sensory ganglia, where they provide rapid and localized immune defense against HSV [8]. These cells are characterized by the expression of canonical residency markers, including CD69 (a retention marker that suppresses tissue egress) and CD103 (αE integrin, which facilitates epithelial adhesion, primarily on CD8^+^ T_RM_). They often express IFN-γ, IL-2, and IL-17, reflecting their cytotoxic and cytokine-producing capabilities. Expression of IL-7Rα (CD127) and responsiveness to IL-15 support their long-term survival and retention. T_RM_ cells actively survey tissues, rapidly eliminate HSV-infected cells, and help maintain local viral control.

**Figure 7:**
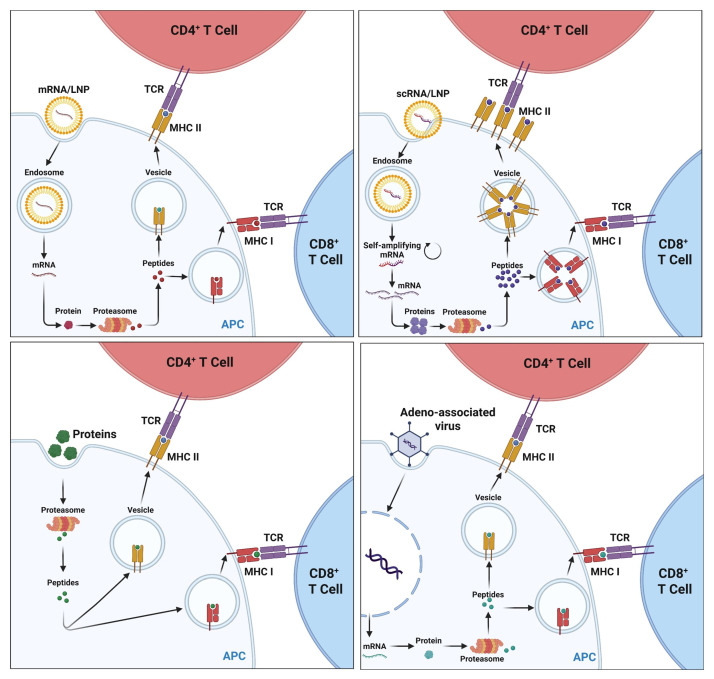
Various HSV vaccine delivery systems and their mechanisms of action. After intramuscular injection, lipid nanoparticle (LNP) encapsulated mRNA is delivered into muscle cells or nearby bystander cells. Once inside, the mRNA is released and translated by ribosomes to produce the viral antigen which is then secreted. Antigen-presenting cells, such as DCs, capture the antigen and initiate an immune response. Adenoviral vector and adeno-associated vaccines deliver viral DNA via adenoviral vectors into muscle or bystander cells. Following uncoating, the DNA tagged with a nuclear localization signal is transported into the nucleus, where it is transcribed into mRNA. The DNA remains extrachromosomal and does not integrate into the host genome. The resulting mRNA is translated into protein, which is secreted and subsequently taken up by the antigen-presenting cells (APCs) to trigger an immune response. Subunit vaccines provide pre-manufactured viral proteins directly to the body. Resident APCs, particularly dendritic cells, internalize the protein and initiate immune activation. The inclusion of adjuvants further enhances the immune response.

HSV-1 and HSV-2 share ~83% of nucleotide identity, and cross-protective immunity may be achieved due to the extensive repertoire of cross-protective antigens [99]. To this end, novel live-attenuated vaccine strategies are being implemented to tame the virus in vivo. In the 1970s and 1980s, the first whole-inactivated HSV vaccine approach used “killed” virus after exposure to heat, UV light, or chemicals. Vaccination using HF10, which is a live-attenuated replication-competent HSV-1 naturally mutated for UL43, UL49.5, UL55, and UL56, and latency-associated transcripts, protected mice against clinical symptoms elicited by HSV-2 by inhibiting viral replication at the site of virus introduction, reducing local inflammation and neuroinvasion, and increasing overall survival [100,101]. The protective effect of HF10 was also attributed to the induction of cellular immunity, primarily mediated by Th1 CD4^+^ T cells (Figure 6 and Figure 7) [101]. HSV-GS3 and HSV-GS7 are replication-competent HSV-1 vectors that demonstrated efficacy in the mouse model of dermal HSV-1 infection [102]. Their replication is regulated by placing one or two essential genes under the stringent control of a gene switch coactivated by heat and antiprogestin [102]. These HSV-1 vectors cannot replicate in the absence of these activating factors [102]. In this study, the inactivated HSV-1 vectors offer equivalent protection to chemically inactivated vaccines [102]. However, the activation of these controlled HSV-1 vectors enhances vaccine efficacy compared to inactivated vaccines. The HSV-1 0ΔNLS, which lacks the nuclear localization signal of the viral ubiquitin ligase ICP0, and the non-neurotrophic HSV-1 vaccine vector VC-2 with deletions in the amino terminus of both the gK and UL20 genes, demonstrated adequate protection against ocular HSV-1 challenge [103]. VC2 (Rational Vaccines) is a live-attenuated HSV-1 vaccine engineered to be incapable of entering neuronal axons. Such attempts to minimize side effects via engineering have been labeled rational [104]. VC2, which possesses deletions of gK aa31-68 and UL20 aa4-22, successfully protected against ocular immunopathogenesis in mice while preventing viral entry to neurons [104]. Additionally, intramuscular administration to guinea pigs resulted in a transcriptional profile characterized by Th17 and regulatory Tr1 responses [104]. Mice vaccinated with HSV-1 0ΔNLS showed superior protection against early viral replication, neuroinvasion, latency, and mortality following ocular challenge with a neurovirulent clinical isolate of HSV-1 [105]. Moreover, 0ΔNLS-vaccinated mice exhibited protection against ocular immunopathology and maintained corneal mechanosensory function [105]. However, only humoral immunity was identified as a significant correlate of protection as demonstrated through passive immunization [105]. Vaccinated mice showed suppressed T cell activation in the draining lymph nodes following the challenge, and vaccine efficacy correlated with serum neutralizing antibody titers [105].

### 4.2. Replication-Defective Vaccines

HSV529 (Sanofi Pasteur) or dl529 is a replication-defective HSV-2 mutant with deletions in two essential genes, UL5 and UL29, both of which are required for viral replication [106]. It was shown to elicit both humoral and cell-mediated immunity, as well as serum-neutralizing antibody titers, serum and vaginal antibodies to HSV-2 glycoprotein D, HSV-2-specific antibody-dependent cellular cytotoxicity, and CD4^+^ and CD8^+^ T cell responses [104,106]. Ninety-nine percent of vaccine recipients experienced a mild to moderate injection site reaction, compared to 47% of placebo recipients [106]. A total of 64% of vaccine recipients experienced systemic reactions, compared to 53% of placebo recipients [106]. Two documented serious adverse events in two participants were concluded to be unrelated to HSV529 administration [106]. This construct has demonstrated protective efficacy against primary infection and recurrences in the guinea pig model. In both HSV-1 seropositive and seronegative animals, vaccination with dl5-29 significantly reduced vaginal viral shedding following challenge [106]. However, studies suggest that dl5-29 elicits limited mucosal IgA and tissue-resident memory CD4^+^ and CD8^+^ T cell responses (Figure 6 and Figure 7) [104]. Therefore, live-attenuated HSV vaccines and replication-defective HSV vaccines were later evaluated. Due to weak immunogenicity, only a few replication-defective and live-attenuated vaccine candidates have progressed into clinical trials [107]. In addition, these vaccines carry the risk of regaining their pathogenicity under immunocompromised conditions [107]. To avoid safety issues that may occur with live-attenuated and replication-defective vaccines, protein-based subunit vaccines have been explored.

### 4.3. Protein/Adjuvant Vaccines

Subunit/peptide vaccines are desirable for vaccine development because they are reasonably stable, safe, and potentially effective [108] (Figure 5). Targeting the primary entry mediators of the virus glycoprotein D and glycoprotein B (gD/gB), which are major antigenic determinants, was the primary focus of subunit vaccine development, as these immunogens stimulate highly effective neutralizing antibodies. Additionally, GE has been utilized in subunit vaccines to target cell-to-cell spread and immune evasion [109]. Recombinant HSV-2 glycoprotein D (gD) has been tested in several clinical trials over the past 30 years [110]. In 1994, the first therapeutic vaccine trial used gD with an aluminum salt (i.e., Alum) adjuvant and reduced recurrence frequency by 24%, despite boosting virus-neutralizing antibodies [111]. In 1997, the Chiron vaccine trial utilized a combination of gD and gB, delivered with the MF59 adjuvant, an oil-in-water emulsion of squalene oil [112]. This gB/gD/MF59 vaccine produced high levels of neutralizing antibodies yet had only 9% efficacy [112]. GEN-003 (GEN-003/MM-2) by Genocea contains recombinant HSV antigens, specifically glycoprotein D (gD) and ICP4, along with Matrix M-2 (MM) adjuvant [113]. In pre-clinical studies, Skoberne et al. (2013) demonstrated that GEN-003 induced broad-spectrum immune responses in mice and exhibited therapeutic efficacy in guinea pigs, resulting in a decrease in recurrent shedding [113]. In 2015, the phase 1 and 2 trials of GEN-003 demonstrated significantly reduced genital lesions and viral shedding in over 310 participants [114]. Genital HSV-2 shedding was significantly reduced in all active vaccine groups, with a 60% reduction in the rate of genital lesions and elevated neutralizing antibody titers [114]. Despite such results, Genocea ceased spending on GEN-003, shifting its focus to neoantigen cancer vaccines in 2017 [115]. Another HSV subunit vaccine that failed to progress past Phase 2 is HerpV (by Agenus). HerpV (formerly called AG-707) consists of 32 HSV-2 peptides derived from 22 HSV-2 proteins that are non-covalently complexed to a heat shock protein 70 (HSP70) chaperone and formulated with a QS-21 saponin adjuvant [116]. Peptides for the vaccine, which include proteins spanning all classes of herpes proteins, were selected based on algorithms that predict human leukocyte antigen binding, synthesis feasibility, and proteasomal processing (Figure 7) [117]. Preliminary results from a phase 2 study of HerpV showed a 15% decrease in viral shedding, which persisted up to 6 months after the initial vaccine series [118]. Simplex (by GlaxoSmithKline) is a truncated glycoprotein D2 (gD2) vaccine candidate that was tested in the phase 3 Herpevac Trial for Women [119]. Subjects were vaccinated with either the investigational vaccine (consisting of 20 μg of glycoprotein D2 from HSV-2 strain G in alum and 3-O-deacylated monophosphoryl lipid A as an adjuvant) or a control hepatitis A vaccine [119]. Three doses of the vaccine were 58% protective against culture-positive HSV-1 genital disease but not protective against HSV-2 infection or disease [119]. Ultimately, it was concluded that the vaccine was unsuccessful in preventing HSV-2 infection or disease, as some women who became infected during the trial experienced recurrent disease [119,120].

Over the last 20 years, adjuvanted glycoprotein D has been evaluated as a primary subunit vaccine in numerous clinical trials, but it has failed to meet the primary endpoint of reducing recurrent herpes disease, despite inducing systemic HSV-specific CD4^+^ Th1 cell responses, as discussed above. These failures emphasize the need to identify new antigens and introduce an innovative tissue-targeted vaccine strategy that induces local T cell immunity in the TG, the site of HSV-1 latency and reactivation cycles [22]. Ruchi et al. investigated the protective therapeutic efficacy of subunit vaccine candidates based on eight recombinantly expressed HSV-2 envelope and tegument proteins in a guinea pig model of recurrent genital herpes [92]. These viral protein antigens (Ags) were rationally selected for their ability to elicit strong CD4^+^ and CD8^+^ T cell responses in naturally “protected” asymptomatic individuals, who, despite being infected, never develop recurrent herpetic disease [92]. Out of the eight HSV-2 proteins, the envelope glycoprotein D, the tegument protein VP22, and the ribonucleotide reductase subunit two protein produced significant protection against recurrent genital herpes [92]. Subunit vaccines often fail to elicit robust cellular immunity, particularly tissue-resident CD8^+^ and CD4^+^ T cell responses at mucosal and ganglionic sites where HSV establishes latency and reactivation. Despite promising pre-clinical data and strong antibody responses, HSV subunit vaccines have consistently failed to meet clinical endpoint criteria. The narrow antigenic focus of these vaccines may limit their effectiveness, as they do not account for the full spectrum of viral proteins involved in immune evasion, latency, or reactivation. A key limitation associated with subunit vaccines is that they may not effectively induce durable memory responses without the use of potent adjuvants or delivery systems. The use of adjuvants may cause local or systemic reactogenicity and induce unintended immune activation. In addition, the inability to stimulate robust T cell-mediated immunity further compromises their ability to control viral latency and prevent recurrent disease, emphasizing the need for alternative vaccine strategies that elicit broad and durable cellular and humoral responses.

### 4.4. DNA Vaccines

DNA vaccines use engineered plasmid DNA to encode one or more viral antigens to stimulate an immune response. DNA vaccines represented an approach for herpes simplex virus immunization due to their ability to induce both humoral and cellular immune responses, stability during storage, and ease of manufacturing. In fact, a DNA vaccine expressing a pool of HSV-2 glycoproteins (gB2, gC2, gD2, gE2, gH2, gL2, and gI2) adjuvanted with IL-12 outperformed the gD2 subunit vaccine [99]. Pre-clinical studies in animal models have demonstrated that DNA vaccines encoding key HSV glycoproteins such as gB, gC, gD, gE, and gH can elicit strong protective immunity [99]. In murine models, DNA vaccines encoding gD have consistently demonstrated the ability to generate neutralizing antibodies and robust CD4+ and CD8+ T cell responses, resulting in reduced viral replication, decreased disease severity, and prolonged survival after challenge with HSV-1 or HSV-2 (Figure 7) [99]. In guinea pig models of recurrent genital herpes, plasmid vaccines expressing gD, in combination with adjuvants like IL-12 or chemokine fusions, reduced lesion recurrence and viral shedding [121]. One of the most notable clinical-stage DNA vaccines is COR-1, developed by Coridon (later Vaxine Pty Ltd.), is a DNA vaccine consisting of two plasmids [122]. One (codon optimized) codes for the HSV-2 envelope glycoprotein D (gD2), and the second has a truncated gD2 fused to ubiquitin [122]. In a pre-clinical model, Cor-1 induced a balanced adaptive humoral and cell-mediated immune response in mice. A phase 1 dose-escalating study showed safety and tolerability in 20 subjects [122]. SL-V20 (by SL VAXiGEN), a plasmid DNA vaccine against HSV2 glycoproteins gC, gD, and the UL39 ribonucleotide reductase, was 100% effective against mouse lethal challenge while also completely preventing vaginal infection [123]. SL-V20 effects were T cell-mediated, with B cells being dispensable to responses [123]. The current status of this vaccine is unclear. VCL-HB01 (Vical), another therapeutic vaccine candidate that was recently abandoned, was VCL-HB01, a DNA plasmid vaccine consisting of polynucleotides encoding codon-optimized gD2 and VP11/12 in combination with Vaxfectin, a lipid-based compound designed to enhance protein expression [104]. A phase 2 study conducted on 261 healthy HSV-2-seropositive adults with a self-reported history of at least 4 to 9 yearly recurrences did not meet its primary endpoint of reducing lesion recurrence rates despite the absence of serious adverse effects [104]. Hence, VCL-HB01 represents another vaccine candidate targeting HSV glycoproteins that failed to progress after unsatisfactory phase 2 results.

While DNA vaccines have shown promise in inducing immune responses, their limited immunogenicity and suboptimal protection in clinical settings highlight the need for improved vaccines and strategies. Challenges with DNA vaccines include relatively low immunogenicity in humans compared to live or viral-vectored vaccines, which has prompted the use of electroporation and novel delivery vectors (e.g., nanoparticles or lipid-based systems) to enhance uptake and expression. DNA vaccines are safe and stable; however, their limited delivery to the nucleus, poor immunogenicity in humans, and complex delivery methods make them less effective. Therefore, a better strategy is necessary to inform B and T cell-targeted vaccines, emphasizing not only the magnitude but also the localization and quality of the immune response, for long-term protection.

## 5. Lessons Learned from Past Genital Herpes Vaccine Clinical Trials

Four vaccine approaches have been tested in the past four decades to fight herpes infections and diseases [124]: (1) inactivated “killed” HSV vaccines; (2) live-attenuated HSV vaccines; (3) replication-defective HSV vaccines; and (4) subunit HSV vaccines (Table 1 and Figure 5). In the 1970s and 1980s, the first whole-inactivated HSV vaccine approach utilized “killed” virus after exposure to heat, UV light [125], or chemicals [126,127]. While inactivated HSV vaccines induce antibodies, they fail to induce robust T cells, and as such, have not been successful in protecting against recurrent genital herpes [128,129]. Therefore, live-attenuated HSV vaccines [130,131] and replication-defective HSV vaccines were subsequently introduced [132]. Due to their weak immunogenicity and safety concerns, only a few replication-defective and live-attenuated vaccine candidates have progressed to clinical trials [107]. A complication of these vaccines is that they carry the risk of regaining their pathogenicity in immunocompromised patients [107]. To avoid potential safety issues that may occur with live-attenuated and replication-defective vaccines, protein-based subunit vaccines have been explored. Recombinant HSV-2 glycoproteins D and B (gD and gB) have been tested in several clinical trials over the past 20 years [110,114,133]. In 1994, the first therapeutic vaccine trial used gD with an aluminum salt (i.e., Alum) adjuvant, followed by the Chiron vaccine in 1997, which employed a combination of gD and gB, generating high levels of neutralizing antibodies [111,134]. Later, two GlaxoSmithKline (GSK) vaccine trials, in 2004 [135] and 2012 [7], used the gD protein delivered with the potent adjuvant, containing 3′-0-deacylated monophosphoryl lipid A (MPL) and a TLR4 agonist, together with Alum [135]. Genocea’s GEN-003 vaccine combined HSV-2 antigens (gD2 and gB2) with the Matrix-M2 adjuvant [114]. However, these HSV subunit vaccines have not achieved their primary outcome. Currently, two ongoing herpes vaccine clinical trials have started in 2023–2024 [55,136,137,138,139,140,141]. Both trials are using conventional base-modified mRNA/LNP technology to deliver a combination of three glycoproteins (gC, gD, and gE): one by Moderna, known as the mRNA-1608 vaccine, and the other by Pfizer/BioNTech, referred to as the BNT-163 vaccine. The results from these mRNA-based herpes vaccine clinical trials are not yet available. These failures underscore the need for an innovative tissue-targeted immunotherapeutic strategy that induces local T cell immunity in the ganglia, the sites of HSV latency and reactivation.

The results of previous clinical trials emphasize four prerequisites for a successful genital herpes therapeutic subunit vaccine: (1) Antigen selection is critical, and targeting a single glycoprotein, such as gD, has proven insufficient for comprehensive protection. Multivalent strategies should incorporate multiple viral proteins and herpes T cell Ags, rather than just B cell Ags (e.g., gB, gC, gD, and gE), in a future herpes vaccine [22]; (2) include protective “asymptomatic” Ags and exclude “symptomatic” Ags that may potentially exacerbate genital herpes disease (e.g., the “symptomatic” gK Ag); (3) design a vaccine strategy that boosts antiviral tissue-resident CD4^+^ and CD8^+^ T cell-mediated immunity (in addition to HSV-specific neutralizing antibodies) [22]; and (4) increase both (a) central immunity at latently infected DRG/TG and (b) peripheral immunity at VMC/ocular tissues. Lastly, population differences, including variations in sex, prior HSV exposure, and genetic background, can significantly influence vaccine efficacy, underscoring the need for personalized or population-tailored vaccine strategies. These insights have driven a shift toward next-generation platforms, particularly those based on mRNA and DNA. These technologies offer flexible, multivalent design, strong induction of both antibody and T cell responses, and the potential to elicit mucosal immunity more effectively. Early pre-clinical studies using mRNA vaccines encoding HSV glycoproteins and tegument proteins have shown potent protection in animal models, suggesting that these newer platforms may overcome the limitations seen in earlier trials and bring us closer to an effective vaccine against herpes simplex virus. Table 2 summarizes recent genital herpes therapeutic vaccine clinical trials.

## 6. New Emerging Herpes Vaccine Strategies

### 6.1. Adenoviral Vectors to Deliver “Asymptomatic” Antigens

Adenoviral vectors (AVs) are emerging as powerful delivery platforms for vaccines and gene-based therapies against the herpes simplex virus (Figure 5). They are a relatively new technology, although adenoviruses have been used as gene delivery vehicles since the earliest days of gene therapy [146]. They are an essential therapeutic vector due to their well-defined biology, genetic stability, better transduction efficiency, and ease of mass production [146]. They are characterized by their high immunogenicity, which occurs through both innate and adaptive inflammatory responses against the adenoviral capsid structures [146]. Their broad tissue tropism, expression of the target antigen, and ability to trigger potent immunogenicity have been utilized to create vaccine candidates for cancer immunotherapies, as well as infectious diseases such as Ebola, AIDS, Zika virus, tuberculosis, and malaria [8,147]. Furthermore, the flexible viral biology allows researchers to engineer them to produce vaccines with increased efficacy. Many approved vaccines developed for SARS-CoV-2 were based on immunization by spike-protein-encoding adenovirus vectors [148]. CanSino Biologics developed Convidecia (AD5-nCOV) using an Adenovirus type 5 (Ad5) vector with the genome of the SARS-CoV-2 Spike protein [148]. A Phase I dose-escalation trial was conducted in healthy volunteers, 108 of whom were in the 18–60 age group, and the results showed a safe and tolerable profile [149].

Several pre-clinical studies have investigated adenoviral vectors expressing HSV antigens, including gD, gB, and gC, as well as immune evasion proteins such as gE [150]. Adenoviral vectors, such as Ad5 or chimpanzee-derived ChAdOx1, have demonstrated strong immunogenicity in pre-clinical models by delivering HSV antigens gD2 and gB2, as well as ribonucleotide reductase subunits [151]. These vectors elicit robust CD4^+^ and CD8^+^ T cell responses, as well as tissue-resident memory cells, at mucosal and neural sites, mimicking the immune control observed in asymptomatic HSV carriers. Quadiri et al. investigated the protective therapeutic efficacy of five recombinant adenovirus-based therapeutic vaccine candidates (rAd-Ags), each expressing different HSV-2 envelope and tegument proteins: RR1, RR2, gD, VP16, and VP22 [8]. The authors observed the frequency and function of DRG and vaginal mucocutaneous (VM) resident CD4^+^ and CD8^+^ T cells induced by these adenoviral vaccines and demonstrated high frequencies of DRG and VM tissue-resident IFN-γ-producing CD4^+^ and CD8^+^ T_RM_ cells associated with significant reductions in viral shedding and genital herpetic lesions [8].

Additionally, adeno-associated virus (AAV) vectors, known for their safety and persistence, are being explored for therapeutic approaches to edit the HSV genome (Figure 5). Recent studies using AAV9, AAV-DJ/8, and AAV-Rh10 to deliver meganucleases and chemokines have shown significant reductions in latent HSV DNA in sensory ganglia and decreased viral shedding in mice [152]. Both vector systems are helping to redefine prophylactic and therapeutic vaccine approaches by enabling durable T cell immunity and novel methods for controlling viral latency [152]. However, there are a few challenges, including potential pre-existing immunity to common adenovirus serotypes, which may reduce vaccine efficacy. Newer strategies, such as exploring less common adenovirus serotypes (e.g., Ad26, chimpanzee adenoviruses) or modified vectors, can help overcome this hurdle. Although no adenoviral-based HSV vaccine has yet reached human licensure, continued development, and combination with platforms such as mRNA vaccines or nanoparticle delivery systems could further enhance efficacy against HSV-1 and HSV-2 infections.

### 6.2. Modified mRNA Lipid Nanoparticle (mRNA/LNP) Therapeutic Herpes Vaccines

The success of nucleoside-modified mRNA/LNP vaccines during the COVID-19 pandemic has catalyzed a wave of innovations in the field, broadening the scope of mRNA vaccine applications and refining their design for enhanced protective efficacy against several viral infections (Figure 5) [153,154,155,156]. The synthetic production methods for the modified mRNA vaccine, delivered in lipid nanoparticles (LNPs), elicited both B and T cell responses, thus avoiding anti-vector immunity [153]. The mRNA/LNP vaccines elicited potent antigen-specific T cells as well as antibodies that were capable of neutralizing SARS-CoV-2 [153]. The successful employment of the SARS-CoV-2 mRNA vaccine has accelerated the research and development of other mRNA vaccines against other viral infections, such as influenza [157,158,159,160]. The mRNA/LNP vaccine strategy has been shown to be overall well-tolerated, with few side effects such myocarditis and pericarditis, particularly in younger males after the second dose of an mRNA vaccine, while providing long-lasting B and T cell immunity in humans [153]. Advancements in delivery systems, particularly LNPs, have significantly enhanced mRNA vaccine performance by improving stability, facilitating efficient cellular uptake, and boosting immunogenicity. Furthermore, innovations in nucleotide modifications, 5′ and 3′ UTR engineering, and codon optimizations have significantly improved mRNA stability and translational efficiency.

Additionally, two forms of modified mRNA vaccines have been developed: conventional mRNA vaccines and self-amplifying RNA (saRNA) vaccines [161]. They have significant advantages over other traditional vaccine approaches, including rapid, scalable, acellular, in vitro production of large quantities and doses of the mRNA vaccine that do not require complex infrastructures (such as mammalian cell culture and protein purification systems) [161]. Both conventional mRNA and self-amplifying mRNA cannot integrate into the host genome and are degraded naturally during the process of antigen expression within hours. These characteristics explain why the modified mRNA vaccines are safer than other vaccines and, therefore, represent a new platform for developing a new herpes vaccine [162].

Conventional mRNA/LNP vaccine strategy is also being pursued for herpesviruses [154]. Awasthi et al. showed the efficacy of a trivalent, nucleoside-modified mRNA vaccine in preventing both clinical and subclinical genital HSV-2 disease in mouse and guinea pig models of genital HSV-2 infection [137]. Vaccination prevented the formation of genital lesions in guinea pigs and mice challenged with HSV-2. Additionally, two doses of 10 μg of the trivalent mRNA vaccine outperformed three doses of 5 μg each of the trivalent subunit vaccines. The mRNA vaccination scheme stimulated superior systemic and vaginal HSV-2-specific IgG, neutralizing antibodies, and gD2-specific antibodies [137,141]. This mRNA vaccine demonstrated superior immunogenicity, as evidenced by the stimulation of long-lived CD4^+^ T cells, T follicular helper cells, and germinal center B cell responses [137]. Currently, there are two ongoing herpes mRNA/LNP vaccine clinical trials, which started in 2023–2024. Both trials are using conventional base-modified mRNA technology to deliver a combination of three glycoproteins (gC, gD, and gE): one by Moderna, known as the mRNA-1608-P101 vaccine, and the other by Pfizer/BioNTech, referred to as the BNT-163 vaccine. Moderna, in collaboration with the University of Pennsylvania, has developed mRNA-1608-P101, a trivalent vaccine targeting HSV-2 gC2, gD2, and gE2, which is currently in a Phase 1/2 trial involving 365 patients aged 18 to 55 years (Clinicaltrials.gov ID: NCT06033261). The trial’s duration is from September 2023 to June 2025. In pre-clinical studies, mRNA-1608 provided 100% protection against a lethal challenge [163] while also preventing dorsal root ganglion infection and inducing high titers of neutralizing antibodies and durable responses of CD4^+^ T follicular helper and memory B cells [141,164]. In rhesus macaques, the trivalent mRNA-1608 vaccine induced neutralizing antibodies that blocked gC2 and gE2 immune evasion, stimulated CD4^+^ T cell responses, and elicited 100% protection in a vaginal challenge [141]. Comparison of the mRNA/nanoparticle formulation to baculovirus proteins with CpG/alum revealed that day 2 and 4 vaginal cultures were negative in 23 of 30 (73%) mice in the baculovirus group, compared with 63 of 64 (98%) in the mRNA group [163]. In guinea pigs, 5 of 10 (50%) animals in the trivalent subunit protein group had vaginal shedding of HSV-2 DNA in 19 of 210 (9%) days, compared with 2 of 10 (20%) animals in the mRNA group that shed HSV-2 DNA in 5 of 210 (2%) days (*p* = 0.0052).

Incorporating a broader range of HSV antigens into mRNA-based vaccines holds promise for improving immune coverage and achieving more robust and long-lasting protection against both HSV-1 and HSV-2 infections. In addition to advancements in antigen selection and delivery platforms, novel immunological strategies, such as the Prime/Pull/Keep approach, will further enhance vaccine efficacy by promoting the establishment and maintenance of protective tissue-resident memory T_RM_ cells at mucosal sites. Table 2 summarizes recent therapies for genital herpes in clinical trials.

## 7. The Prime/Pull Vaccine Strategy

Shin & Akiko Iwasaki (2012) introduced and experimentally validated the concept of Prime/Pull in the context of prophylactic HSV vaccines [165]. The Prime and Pull strategy is a two-step immunization approach: (1) conventional parenteral vaccination to elicit systemic T cell responses (Prime) followed by (2) recruitment of activated T cells via topical administration of a T cell attractant (Pull), where such T cells establish long-term protective immunity [165]. A report demonstrated that systemic priming with HSV antigens, followed by topical application of a chemokine (the “Pull”), elicited robust local CD8^+^ T cell responses in mucosal tissues and protected mice from genital HSV challenge [165]. After genital HSV-2 infection, chemokine ligand 9 (CXCL9) and CXCL10 expression are induced by interferon-γ secreted by CD4^+^ T cells and mediate the recruitment of effector CD8^+^ T cells to the infected tissue via the chemokine receptor CXCR3. CXCR3 is expressed by both effector Th1 cells and activated CD8^+^ T cells, as well as other cell types [165].

Following this, the Friedman group extended the application to therapeutic vaccines. It showed that the frequency of recurrent disease and recurrent vaginal shedding was reduced most effectively by the combination of Prime (glycoprotein vaccine) and Pull (vaginal imiquimod) [91]. This concept has also been explored in HIV, where intravaginal chemokine pulls following systemic gp140 vaccination-induced local antibodies and T cell responses [91]. Similar approaches have been tested in *Streptococcus pyogenes* and HPV, where mucosal adjuvants and intranasal chemokines have been shown to enhance immune responses in the respiratory and genital tracts.

While the Prime/Pull strategy has demonstrated success in recruiting effector and memory T cells to the mucosal and neuronal sites of HSV latency and reactivation, it may be insufficient for long-lasting protection. Prime/Pull effectively enhances T cell trafficking to the site of infection. Still, additional strategies are needed to promote local retention, long-term survival, and functional maintenance of these T_RM_ cells [166]. For effective and sustained immunity against HSV, especially in the context of viral reactivation or re-infection, it is critical not only to localize antiviral T cells but also to maintain them in a responsive state over time.

## 8. Next-Generation Prime/Pull/Keep Herpes Vaccine (PPK Vaccine) Strategy

Among the rationales supporting the development of a T cell-based genital and ocular herpes vaccines are the following: (1) T cell immune deficiency predisposes individuals to severe herpes disease. (2) Candidate antigen-based vaccines induce T cell-dependent protection in animal models. (3) HSV-specific T cells can be programmed to traffic to infected sites and persist at sites of HSV-1 and HSV-2 epithelial lytic infection and ganglionic latency. Although the memory CD8^+^ T cell population is heterogeneous in phenotype, function, and anatomic distribution, it can be divided into three major subpopulations: (i) effector memory T cells (T_EM_) that are CD103^low^CD62L^low^CCR7^low^; (ii) central memory T cells (T_CM_) that are CD103^low^CD62L^high^CCR7^high^ [167]; and (iii) tissue-resident memory T cells (T_RM_) that are CD103^high^CD62L^low^CCR7^low^CD11a^high^CD49a^high^CD69^high^ [167,168,169]. In contrast to CD8^+^ T_CM_ cells that must undergo differentiation for effector function [170,171,172,173], the T_RM_ cells are already differentiated and poised for immediate effector function [174]. We recently found that an increased number of HSV-specific CD8^+^ T_RM_ cells expressing high levels of tissue homing and tissue residency receptors (i.e., CXCR3, IL-2R/IL-15R, CD69, and CD103), which reside in the TG of HSV-1-infected HLA-A*0201 transgenic rabbits (HLA Tg rabbits), was associated with decreased virus reactivation in the TG and reduce virus shedding in the cornea [76]. (4) Importantly, antibody-targeting genital herpes vaccines (e.g., gB and gD) have had low or no efficacy in clinical trials.

**Figure 8:**
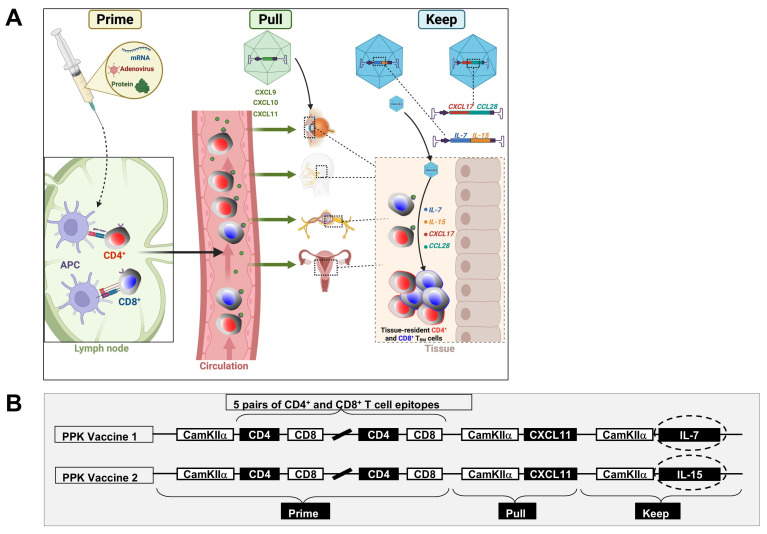
Illustration of the mechanism of action of the Prime/Pull/Keep vaccination strategy using T cell-attracting chemokines and survival cytokines to attract and retain antiviral protective tissue-resident memory T cells within infected tissues: (**A**) A multi-step immunization approach designed to enhance local antiviral immunity in mucosal tissues. First, systemic priming with HSV antigens induces circulating virus-specific T cells. Subsequently, a chemokine-mediated pull is performed using CXCL11, CXCL17, and CCL28, which attract activated T cells to epithelial, mucosal, and neuronal sites. CXCL11 primarily recruits CXCR3^+^ effector T cells, CXCL17 supports recruitment to epithelial surfaces, and CCL28 draws CCR10^+^ cells to mucosal tissues. To promote the long-term retention and survival of tissue-resident memory T cells, IL-7 and IL-15 are administered either locally or systemically. IL-7 supports survival through IL-7Rα signaling, while IL-15 contributes to the maintenance of CD103^+^ CD8^+^ T_RM_ cells. (**B**) A single-step immunization approach using an all-in-one herpes simplex PPK vaccine molecule delivering human CD4^+^ and CD8^+^ epitopes selected from multiple HSV-1 or HSV-2 protein antigens to induce human T cells (Prime), linked to a T cell-attracting CXCL11 chemokine to attract primed T cells into infected ganglia and genital tract epithelial tissues (Pull) and IL-7 and/or IL-15 to maintain primed and pulled T cells for a long time within infected tissues (Keep). CXCL11, IL-7, and IL-15 are expressed under tissue-specific promoters and delivered using tissue-specific AAV vectors.

The CD8^+^ tissue-resident memory (T_RM_) cells are thought to never leave the TG and DRG, like non-lymphoid tissues, where they surveil virus re-infection or reactivation (Figure 6). The CD8^+^ T_RM_ cells express elevated levels of the CD69 C-type lectin receptor and CD103, which is part of the integrin αEβ7, to enforce their tissue retention in peripheral non-lymphoid tissues [175]. However, contrary to the current general perception, in many infection models [176,177], at least some CD8^+^ T_RM_ cell subsets do transiently leave non-lymphoid tissues to enter the circulation. A low frequency of CD8^+^ T_RM_ cells present around latently infected neurons is insufficient to prevent virus reactivation. Therefore, it is possible that, like infectious models, a low frequency of resident CD8^+^ T_RM_ cells surrounding latently infected neurons in symptomatic herpes patients with increased virus shedding may be caused by some CD8^+^ T_RM_ cell subsets migrating out of the neurons to enter the circulation (Figure 6). Therefore, an innovative tissue-targeted Prime/Pull/Keep (PPK) therapeutic vaccine approach, inspired by a previously published study on genital herpes Prime/Pull [54], could be leveraged to both enhance recruitment and promote the long-term retention of antiviral T cells at ganglionic sites (Figure 8). Prime/Pull effectively enhances T cell trafficking to the site of infection. Still, additional strategies are needed to promote local retention, long-term survival, and functional maintenance of these T_RM_ cells (Figure 6). For effective and sustained immunity against HSV, especially in the context of viral reactivation or re-infection, it is critical not only to localize antiviral T cells but also to maintain them in a responsive state over time. This innovative PPK vaccine strategy would “pull” back and “keep” the CD8^+^ T_RM_ cell subsets within the latently infected neurons, which would otherwise leave the infected neurons and contribute to an increased number of resident CD8^+^ T_RM_ cells, reaching a threshold that would efficiently stop or reduce virus reactivation from latently infected neurons. Inducing robust and sustained protection by bolstering and maintaining antiviral tissue-resident effector and memory CD4^+^ and CD8^+^ T cells at mucosal and ganglionic sites by the Prime/Pull/Keep (PPK) therapeutic vaccine strategy would constitute a paradigm shift in the herpes vaccine field. Such a comprehensive “Prime/Pull/Keep” approach would ensure the persistence and readiness of T_RM_ cells at mucosal and ganglionic sites. This may involve strategies such as local cytokine modulation or vaccine boosting to preserve protective T cell niches (Figure 8).

### 8.1. Using CXCL17 and CCL28 Mucosal Chemokines, IL-7, and IL-15 Cytokines in the PPK Therapeutic Vaccines to “Keep” Memory CD4^+^ and CD8^+^ T_EM_ and T_RM_ Cells Within the Ganglia and Peripheral Epithelial Tissues

Pulling more antiviral tissue-resident effector CD4^+^ and CD8^+^ T_RM_ cells within latently infected DRG and VMC of guinea pigs is associated with protection from recurrent genital herpes. Conversely, depletion of functional CD4^+^ and CD8^+^ T cells in the latently infected and vaccinated guinea pigs and mice was associated with increased virus shedding, severity, and longevity of genital herpes. Chemokines are proteins that induce chemotaxis and promote T cell homing into infected tissues [178]. While there are 49 chemokines [178], two VMC-specific mucosal chemokines, CCL28 and CXCL17, are specifically expressed in the vaginal mucosal tissue in homeostasis and induced following herpes infection of the genital tract [179,180]. In addition, the phenotypic and transcriptomic profiling of symptomatic and asymptomatic individuals has shown that frequent HSV-specific CD4^+^ and CD8^+^ T_RM_ cells, with elevated expression of CXCR3 (the receptor of CXCL11), CXCR8, and CCR10 (the receptor of CXCL17 and CCL28 mucosal chemokines), were associated with protection [84,180]. CCL28 and CXCL17 guide, attract, and relocate specific subsets of CD4^+^ and CD8^+^ T cells within mucosal tissues infected with pathogens. CCL28, initially discovered, was found to bind to CCR10 and is highly expressed on mucosal epithelial cells [181,182] (Figure 8). The CCL28 chemokine enhances protection against genital herpes by mobilizing antiviral effector memory cells into the infected vaginal mucosa [180]. In addition, Zlotnik et al. were also the first to report that (1) CXCL17 is a mucosal chemokine and (2) CXCL17 signals through the orphan G protein-coupled receptor-35 (GPR35), also known as CXCR8 [183]. CXCL17 chemokine-dependent mobilization of effector memory and tissue-resident memory T cells in the vaginal mucosa is associated with protection against genital herpes [179]. Accordingly, CXCL17^(−/−)^ and CCL28^(−/−)^ deficient mice lost protection against genital herpes [179,180].

Consistent with the above discussion, maintaining frequent antiviral tissue-resident memory CD4^+^ and CD8^+^ T_RM_ cells within the latently infected DRG and VMC is required for sustained, long-term protection from recurrent genital herpes [84]. Cytokines such as interleukin-7 (IL-7/IL-7R ligand) and interleukin-15 (IL-15/IL-15R ligand) are widely considered necessary for the maintenance or “keeping” of memory CD8^+^ T cells [184] (Figure 8). IL-15 plays a critical role not only in the homeostatic proliferation of circulating memory CD8^+^ T cells but also in the development, survival, and functional maintenance of tissue-resident memory T cells [185]. IL-15 is also required for the development and maintenance of CD103^+^ CD8 T_RM_ in the skin epidermis following HSV infection [186] (that can produce multiple effector molecules and cytokines, which enhance their ability to control the virus (Figure 6 and Figure 8). IL-15 signaling supports the metabolic fitness and antiapoptotic profile of these cells, enabling them to remain stably lodged within epithelial tissues without recirculating [187]. It promotes local survival cues via trans-presentation by IL-15Rα-expressing dendritic cells and keratinocytes [188]. This mechanism is fundamental in non-lymphoid tissues, such as skin (Figure 6 and Figure 8). Furthermore, IL-15-driven signaling contributes to the expression of hallmark T_RM_-associated molecules, including CD69 and CD103, which anchor these cells in peripheral tissues and prevent their migration out of these tissues. Using inducible deletion of IL-7Rα in mature memory T cells, researchers have demonstrated that IL-7Rα is essential for the ongoing, low-level proliferation of memory CD8^+^ T cells [189].

In contrast, IL-15 appears to be more crucial for maintaining the resident memory pool by promoting survival and retention within barrier tissues (Figure 8). The loss of IL-7Rα caused a gradual decline in memory T cells across various tissues [190]. The primary effect of losing IL-7Rα was not increased cell death, but somewhat reduced basal (homeostatic) proliferation of memory CD8^+^ T cells [190]. This contrasts with the earlier belief that IL-7’s role was primarily in cell survival. While IL-7Rα-deficient memory cells still responded to antigen re-exposure and were able to proliferate and produce granzyme B, they did not expand as effectively as IL-7Rα-sufficient cells during recall [189] (Figure 8). Therefore, the “PPK therapeutic herpes vaccine,” when engineered to incorporate chemokines such as CCL28 and/or CXCL17, or cytokines such as IL-7 and IL-15, might “keep” or retain large numbers of antiviral memory tissue-resident T_EM_ and T_RM_ cells within the VMC of HSV-2-infected guinea pigs. However, further research is warranted to validate and elucidate this and the mechanisms of this immune retention in vivo.

### 8.2. Using IL-7 and IL-15 Survival Cytokines to “Keep” More Memory CD4^+^ and CD8^+^ T_EM_ and T_RM_ Cells in Latently Infected Trigeminal Ganglia and Cornea

For ocular HSV-1 infection, a similar tissue-targeted immunotherapeutic approach may enhance local antiviral immunity in the TG and corneal tissue. Studies using humanized rabbit models have demonstrated that vaccination with HSV-1-derived peptides identified from asymptomatic HSV-1-positive individuals, followed by CXCL10 chemokine-mediated T cell recruitment into the cornea, conferred protection against ocular HSV-1 challenge [73]. This strategy successfully increased local CD8^+^ T cell infiltration and reduced disease severity. Moreover, asymptomatic individuals with ocular HSV-1 have been shown to harbor functional HSV-specific CD8^+^ T cells in the TG, suggesting that local antiviral T_RM_ cells are critical for immune control and preventing reactivation. Previous studies have demonstrated that HLA-Tg rabbits with fewer TG-resident CD8^+^ T_RM_ cells shed more viruses, and vice versa [191,192]. In addition, our lab has also demonstrated that phenotypic and transcriptomic profiling of TG-resident CD8^+^ T_RM_ cells in asymptomatic HLA Tg rabbits with reduced virus shedding reveals elevated expression of CD69 and CD103 receptors, which are associated with tissue homing and tissue residency. Khan et al. also demonstrated the efficacy of vaccinating humanized rabbits with HSV-1-derived peptides identified in asymptomatic HSV-1-positive individuals, followed by chemotactically pulling immune cells into the cornea using AAV8 expressing CXCL10 chemokine [73]. More recently, Chentoufi et al. demonstrated that a Prime/Pull/Keep AAV8-based vaccine strategy, designed to both “prime” functional antiviral CD8^+^ T cells in peripheral tissues and “pull” and “keep” them in the infected cornea and TG, leads to a significant reduction in corneal HSV-1 infection and disease [76]. Consistent with the above discussion, the long-term control of recurrent ocular infection requires the sustained presence of antiviral tissue-resident memory CD4^+^ and CD8^+^ T (T_RM_) cells within sites of viral latency and reactivation, including the TG and cornea [44]. IL-7 and IL-15, through their respective receptors (IL-7R and IL-15R), are well-established as critical factors for the maintenance and functional persistence or “keep” of memory CD8^+^ T cells [184] (Figure 8). To “keep” CD8^+^ T_RM_ cells for the long term within the latently infected neurons, the PPK vaccine could include survival IL-7/IL-15 cytokines. Therefore, this “Prime/Pull/Keep” vaccination strategy is an innovative approach to vaccine administration that can demonstrate therapeutic efficacy in the most stringent animal models of HSV disease by boosting the quantity, persistence, and cytolytic function of CD4^+^ and CD8^+^ cells in the TG and corneal tissue. PPK therapeutic vaccine would contribute to increasing the number of TG-resident CD4^+^ and CD8^+^ T_RM_ cells to a threshold that would efficiently interfere with virus reactivation from latently infected neurons of the TG. Ultimately, the ability to boost the recruitment of T cells and establish resident T cell populations in peripheral tissues that restrict lymphocyte homing will aid not only in the prevention but also in the treatment of a wide variety of diseases.

A similar strategy, described in malaria vaccine studies, is known as the prime–boost method. This approach involves a systemic prime (using peptide-pulsed dendritic cells) followed by a liver-targeted “trap” using AAV vectors expressing the antigen, effectively capturing antigen-specific CD8^+^ T cells within the liver and generating protective liver-resident T_RM_ cells [108,193]. Beyond viral infections, the “Prime/Pull/Keep” approach is also being applied to improve the recruitment of immune cells to other restrictive microenvironments, such as tumors. Effective immunotherapy can be hindered by either decreased or inappropriate expression of chemokines in the tumor tissue, leading to minimal migration of immune cells. Delivery of appropriate chemokines to the infection site after immunization could enhance recruitment of specific T cells and augment the efficacy of immunotherapies [194].

## 9. Integrating Artificial Intelligence and Deep Learning into Herpes Simplex Virus Vaccine Design

Artificial intelligence (AI) and deep learning (DL) are increasingly recognized as transformative tools in the rational design of next-generation vaccines, particularly for persistent viral infections such as herpes simplex virus (HSV). Within the tissue-targeted Prime/Pull/Keep (PPK) strategy for therapeutic HSV vaccination, AI technologies enhance multiple stages of the vaccine development pipeline, including antigen selection, immunogenicity prediction, and host-pathogen interaction modeling [195]. By leveraging large-scale viral sequence and immunological datasets, DL models can identify conserved, immunodominant B and T cell epitopes across HSV-1 and HSV-2 proteins such as glycoprotein D (gD), glycoprotein B (gB), ICP0, ICP4, RR1, and RR2 prioritizing epitopes capable of stimulating tissue-resident memory T cells (T_RMS_) within mucosal tissues, which are essential for controlling HSV latency and reactivation [195,196].

Moreover, AI-based frameworks that incorporate uncertainty quantification techniques (e.g., Monte Carlo dropout and Bayes-by-backpropagation) provide probabilistic confidence scores alongside immunogenicity predictions, improving the reliability and translational value of selected vaccine candidates [197]. Explainable AI methods, including attention mechanisms and saliency maps, can further reveal key residue-level features that drive epitope immunogenicity, enabling a transparent and hypothesis-driven immunogen design process [195,197]. Generative models such as generative adversarial networks (GANs) and variational autoencoders (VAEs) have also been applied to engineer synthetic, multi-epitope constructs optimized for major histocompatibility complex (MHC) binding and T_RM_ activation, core goals of the PPK framework [198].

One approach that can be used to operate these technologies is a multimodal AI-guided analytic framework that integrates high-dimensional immune profiling datasets, including flow cytometry, epitope-specific tetramer staining, cytokine profiling (e.g., IL-7, IL-15, CXCL11), and single-cell RNA sequencing (scRNA-seq). These data are used to identify phenotypic and transcriptional signatures of protective T_RM_ cells within the trigeminal ganglia (TG), helping to elucidate immune correlates of reduced HSV-1 reactivation and viral shedding [194,199]. Unsupervised clustering and trajectory inference techniques such as UMAP, FlowSOM, and pseudo-time analysis have been applied to define dynamic T_RM_ cell states, particularly under immune checkpoint modulation (e.g., PD-1, TIM-3), offering insight into T cell re-invigoration [200,201,202]. In parallel, explainable AI tools such as SHAP values and attention-based interpretability help identify transcriptional programs associated with durable T_RM_ protection [203,204].

**Figure 9:**
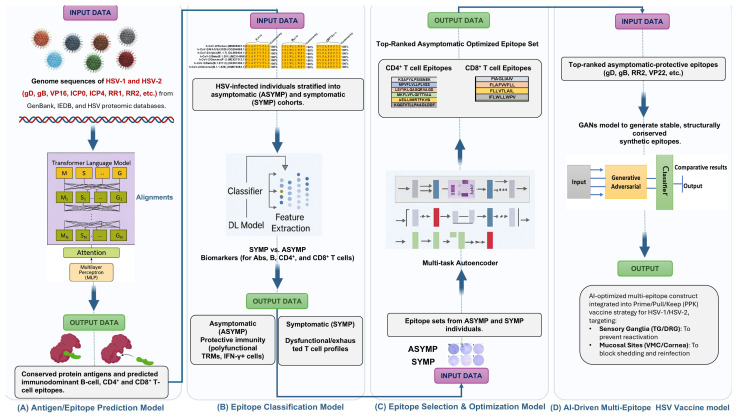
The AI-powered vaccine pipeline will predict conserved and immunodominant epitopes. A deep learning classifier model would identify immune correlates of protection by comparing the immune features of symptomatic and asymptomatic individuals infected with HSV. A multi-task autoencoder would select an optimized epitope that stimulates polyfunctional T_RM_. The epitopes identified would be incorporated into AI-optimized multi-epitope constructs designed to target sensory ganglia and mucosal tissues for HSV-1/HSV-2 immunotherapy.

As illustrated in Figure 9, the AI-powered vaccine pipeline begins with transformer-based language models applied to genome sequences of HSV-1 and HSV-2 (Figure 9A). These models predict conserved and immunodominant B cell, CD4^+^, and CD8^+^ T cell epitopes using inputs from GenBank, IEDB, and HSV proteomic databases. In the next phase (Figure 9B), a deep learning classifier model distinguishes between the immune features of symptomatic (SYMP) and asymptomatic (ASYMP) individuals infected with HSV, enabling the identification of immune correlates linked to protection versus pathology. In Figure 9C, a multi-task autoencoder is used to select optimized epitope sets derived from ASYMP and SYMP datasets, emphasizing epitopes that stimulate polyfunctional T_RM_s. Finally, in Figure 9D, GANs are employed to generate stable, structurally conserved synthetic epitopes, which are then incorporated into AI-optimized multi-epitope constructs designed to target sensory ganglia and mucosal tissues for HSV-1/HSV-2 immunotherapy.

This integrated pipeline enables real-time refinement of immunogen composition (e.g., epitope and cytokine selection), optimization of delivery vectors (e.g., AAV8 scheduling), and pre-clinical down-selection of lead PPKIm candidates. Beyond accelerating the development process, this AI-based decision-support framework reduces reliance on exploratory animal testing and enhances the translational potential of PPK vaccines for neurotropic infections [205].

Based on these computational and immunological strategies, predictive modeling supports the identification of lead PPKIm candidates likely to increase the abundance and longevity of HSV-specific CD4^+^ and CD8^+^ T_RM_ cells within latently infected TG tissue. Reductions in viral reactivation (as measured in TG explant assays) and viral shedding in tears represent two clinically meaningful endpoints that are difficult to achieve with systemic therapies alone. AI-based prioritization can streamline the evaluation of the eight designed PPKIm constructs, enabling selection of those most likely to achieve durable control of HSV latency and transmission (Figure 9).

### 9.1. AI-Driven Epitope Prediction for Targeted Immunity

A critical component of developing a therapeutic vaccine against recurrent herpes simplex virus (HSV) is the precise identification of epitopes that can elicit robust tissue-resident memory T cell (T_RM_) responses within mucosal sites and sensory ganglia, the primary reservoirs of viral latency and reactivation. Artificial intelligence (AI) and deep learning (DL) technologies now offer powerful capabilities to accelerate this process by accurately predicting immunogenic B cell and T cell epitopes across the HSV-1 and HSV-2 proteomes. These computational frameworks not only reduce reliance on labor-intensive experimental screening but also enable integration of host-pathogen data to guide immunogen selection.

Advanced state-of-the-art epitope prediction tools such as NetMHCpan, DeepHLApan, and MARIA (MHC Analysis with Recurrent Integrated Architecture) have been trained on large datasets of peptide HLA-binding affinities, enabling the identification of conserved viral epitopes with high precision across diverse HLA backgrounds [206,207]. The Immune Epitope Database (IEDB) further enhances this process by offering access to experimentally validated T cell epitopes, which can be used to fine-tune model training and cross-validate predictions. These tools are especially valuable in the context of HSV vaccine design, where the goal is to induce T_RM_ cells that persist at sites of potential viral reactivation, providing durable and localized immune control.

Importantly, these AI platforms can be tailored using immunological data from both symptomatic and asymptomatic individuals. Asymptomatic HSV carriers are known to maintain polyfunctional T_RM_ populations capable of suppressing viral shedding without clinical symptoms [208,209]. By incorporating such datasets, AI models can be trained to favor epitopes linked to protective immunity rather than inflammatory or ineffective responses. This capability is highly relevant to therapeutic vaccine strategies aimed at restoring effective immune surveillance in individuals with frequent HSV reactivation. Moreover, the integration of AI into the Prime/Pull/Keep (PPK) framework provides a mechanistic foundation for each phase. During the “Prime” phase, AI-assisted epitope selection enables activation of broad T cell repertoires with the potential to differentiate into T_RM_ cells. In the “pull” phase, predictive models help identify chemokine-inducing sequences that facilitate the recruitment of T_RM_ cells to mucosal surfaces. For the “Keep” phase, epitopes associated with long-lived T_RM_ persistence can be prioritized to sustain antiviral protection over time. Viral antigens such as gB, gD, ICP0, and ICP4, which are well-conserved and play roles in viral entry and immune evasion, are promising sources of such epitopes and can be systematically analyzed through these AI-driven platforms [210,211].

Finally, AI-based population modeling supports precision vaccine design by accounting for the diversity of HLA allele frequencies across global populations [207,212]. This approach ensures equitable vaccine efficacy, particularly important for herpesvirus infections that affect individuals across all demographic groups. Taken together, AI-guided epitope prediction provides a scalable, personalized, and mechanistically grounded foundation for designing next-generation HSV vaccines that align with the immunological principles of the PPK strategy and the goal of long-term control of recurrent disease.

### 9.2. Deep Learning Models for Multi-Epitope Vaccine Design

Designing an effective therapeutic vaccine against the herpes simplex virus (HSV) extends beyond identifying individual epitopes. It requires assembling these epitopes into multi-epitope constructs that are immunologically potent, structurally stable, and capable of inducing durable responses at sites of viral latency. Deep learning (DL) has emerged as a transformative tool in this space by enabling the rational design of such constructs through data-driven simulations of sequence, structure, and immunogenicity.

Generative DL models, including variational autoencoders (VAEs) and generative adversarial networks (GANs) (Figure 9), are increasingly used to create synthetic antigenic constructs that integrate multiple B cell and T cell epitopes derived from HSV proteins such as gD, gB, ICP0, and ICP4. These models are trained on datasets containing known viral antigens, MHC-binding profiles, and protein structures to generate candidate sequences optimized for high MHC affinity, proteasomal cleavage, and epitope clustering [213,214]. This design logic directly supports the PPK vaccine framework, which aims to prime and maintain T_RM_ cells in mucosal tissues and sensory ganglia.

A significant advantage of DL-based epitope assembly is its ability to simulate three-dimensional protein folding and assess the accessibility of epitope surfaces. Proper folding is critical to ensure that immunodominant epitopes are exposed to immune surveillance and are not masked by neighboring regions. DL-powered structure prediction algorithms, such as AlphaFold and RoseTTAFold, have enabled accurate modeling of folding patterns, informing the design of linker sequences that preserve epitope orientation and minimize immunological interference [215,216]. This approach helps maximize antigen presentation and T cell activation at the tissue level, which is essential for controlling HSV latency and recurrence. Furthermore, DL frameworks can rank epitopes based on predicted immunodominance and potential neoepitope formation, minimizing off-target responses or autoimmunity. This computational screening process accelerates pre-clinical development by reducing the reliance on wet-lab optimization and allows for iterative refinement of vaccine constructs. Importantly, population-scale modeling tools can incorporate HLA allele frequencies and regional variations to ensure broad vaccine applicability across genetically diverse populations [216]. This is especially relevant for therapeutic vaccines targeting chronic infections, such as HSV, where variability in immune responsiveness across individuals has historically hindered universal vaccine success.

In essence, deep learning models provide a highly scalable and customizable platform for constructing multi-epitope vaccines. They support the development of vaccine candidates that not only carry the appropriate antigenic targets but also present them in a manner optimized for immunogenicity, structural integrity, and tissue-specific immune programming. These capabilities are critical for enabling the Prime, Pull, and Keep phases of TRM-based HSV immunotherapy.

### 9.3. Modeling T_RM_ Cell Recruitment and Retention Using AI

A cornerstone of the Prime/Pull/Keep (PPK) strategy for therapeutic herpes simplex virus (HSV) vaccination is the induction and maintenance of tissue-resident memory T cells (T_RM_) at the sites of HSV latency and reactivation, such as the vaginal mucosa and sensory ganglia. AI provides a robust framework for modeling and optimizing this process by simulating how specific cytokine and chemokine environments influence T_RM_ cell migration, residency, and longevity within these tissues.

AI-based immune modeling tools integrate high-dimensional data from transcriptomics, proteomics, and single-cell RNA sequencing to predict how molecular signals like CXCL10, CCL27, CCL28, and CXCL17 regulate the recruitment of CD8^+^ and CD4^+^ T cells to infected tissues [217,218]. These chemokines have been implicated in guiding lymphocyte homing to epithelial and neuronal sites, which is crucial for establishing mucosal immunity. Deep learning models trained on these datasets can simulate temporal dynamics of chemokine gradients and their interaction with T cell receptors and integrins to forecast the efficiency of the “pull” phase of the vaccine strategy. Beyond recruitment, AI models can predict the impact of cytokines such as IL-7, IL-15, and TGF-β on the retention and functional programming of T_RM_ cells within mucosal barriers and ganglia. These cytokines are known to support T_RM_ survival and effector function, and their inclusion as adjuvant components can be guided by in silico screening. Systems biology approaches powered by machine learning can identify the most promising molecular combinations that enhance T_RM_ density and functionality without inducing systemic inflammation [219]. This predictive capacity enables rational adjuvant selection and delivery route optimization, reducing the trial-and-error approach traditionally used in vaccine development.

Notably, the AI-driven simulation of T_RM_ behavior under various immunization protocols allows for pre-clinical testing of vaccine schedules and formulations. For example, models can compare different timing intervals for prime and boost phases, or assess whether intravaginal, subcutaneous, or mucosal routes best support T_RM_ establishment in HSV-infected tissues. These computational predictions can be validated in animal models and subsequently refined to improve translational relevance.

AI also supports the personalization of vaccine strategies by modeling individual variation in chemokine receptor expression, tissue microenvironment, and immune history. This is particularly relevant for HSV, as patients exhibit heterogeneity in disease severity, immune responsiveness, and TRM maintenance. By incorporating patient-specific variables, AI-guided models can help tailor PPK-based vaccine strategies for optimal long-term protection against recurrence and viral shedding [220]. Moreover, AI-enabled modeling of chemokine signaling, and cytokine-mediated retention provides an essential toolset for advancing T_RM_-based HSV vaccine strategies. These technologies support the “Pull” and “Keep” phases of the PPK framework by enabling precise, tissue-targeted immune programming that promotes localized, durable immunity at sites critical to HSV pathogenesis.

### 9.4. Expanding the Role of AI and Deep Learning in Prophylactic and Therapeutic HSV Vaccine Design

Artificial intelligence (AI) and deep learning (DL) technologies have revolutionized multiple domains of biomedical research. They are now poised to significantly accelerate both prophylactic and therapeutic vaccine development against herpes simplex virus (HSV). While much of the current focus centers on therapeutic vaccine strategies, especially those aimed at controlling viral latency and reactivation through tissue-resident memory T cells (T_RM_s), AI and DL also offer powerful frameworks for designing prophylactic vaccines that can preempt initial infection and reduce viral spread in the population.

In the development of prophylactic vaccines, AI models can be trained on viral genomic data to identify early-expressed, highly conserved antigens critical for viral entry and replication, such as glycoprotein D (gD), glycoprotein B (gB), and glycoprotein H (gH) [221]. These proteins are primary targets for neutralizing antibodies and CD4+ T cell responses. DL models, such as NetMHCpan and transformer-based architectures, can predict class I and class II MHC-binding affinities for these antigens, enabling rapid identification of epitopes with the highest protective potential [206]. By simulating naive immune responses, these tools help select epitope combinations likely to induce sterilizing immunity, which is a critical goal in prophylactic vaccination [222]. Moreover, DL-driven protein structure prediction and antigen design, enabled by generative adversarial networks (GANs) and variational autoencoders (VAEs), allow for the creation of synthetic immunogens that mimic native viral structures while being optimized for both immunogenicity and safety [223]. This is particularly valuable in designing multivalent vaccines that target both HSV-1 and HSV-2, increasing coverage across viral subtypes. These models also aid in assessing off-target risks, minimizing the chance of neoepitope generation that could lead to unintended autoimmune responses [215].

On the therapeutic side, AI tools go beyond epitope selection to support the Prime/Pull/Keep (PPK) vaccine strategy. In this approach, AI models are utilized to simulate the performance of various antigens, not only in the peripheral priming of T cells but also in promoting T_RM_ recruitment and maintenance in the mucosa and sensory ganglia [165]. Modeling cytokine and chemokine networks, such as IL-7, IL-15, CXCL9, CXCL10, and CCL28, allows researchers to identify optimal molecular adjuvants or delivery strategies to support durable T_RM_ formation [224]. This is essential for suppressing viral reactivation, especially in patients with recurrent HSV.

AI will ultimately validate epitopes based on their immunogenicity and protective efficacy. For the PPK vaccine strategy, AI can integrate multi-omics, structural, and immunoprofiling data to identify phase-specific epitopes, those optimized for priming to induce strong central and peripheral immunity (Prime), those optimized for recruiting protective T_RM_/T_EM_ cells with appropriate homing receptors in response to T cell-attract ting chemokines (Pull), and those optimized for maintaining functional T_RM_/T_EM_ cells with CD69/CD103 and survival signaling pathways (Keep). This AI-assisted selection ensures that each PPK phase is addressed effectively, contributing to optimal protective immunity against herpes infection and disease.

In both contexts, population-scale modeling is key. AI models can incorporate global HLA allele distribution and immune response variability to ensure that vaccine candidates are broadly effective across genetically diverse populations, addressing the historical challenge of inconsistent efficacy in different ethnic or geographic groups [225]. Personalized vaccine design is further supported by integrating individual immune history, tissue-specific expression patterns, and microbiome data to predict how a given patient will respond to specific immunogens [226].

In summary, AI and DL serve as unifying technologies that bridge preventive and therapeutic strategies in HSV vaccine design. Their use directly supports the goals of the PPK vaccine framework by enabling optimized priming, targeted T_RM_ cell recruitment, and long-term retention. These tools are critical for developing both prophylactic and therapeutic HSV vaccines that are safe, effective, and tailored to the biological complexity of HSV latency and recurrence.

## 10. Conclusions

Over the past 25 years, efforts to develop a herpes simplex subunit vaccine have explored multiple antigens, various delivery systems, and adjuvants, yet without success. These failures underscore the need to move beyond just trying antigens, antigen delivery systems, adjuvants, and routes of systemic/parenteral administrations and instead explore innovative tissue-targeted vaccine strategies that would induce or boost local T cell immunity at the mucocutaneous tissues (peripheral immunity) and the ganglia, the sites of HSV latency and reactivation (central immunity).

The above literature supports the rationale for innovative tissue-targeted PPK therapeutic herpes vaccines, specifically designed to boost the number, longevity, and function of CD4^+^ and CD8^+^ T_RM_ cells in ganglionic and mucosal sites, thereby strengthening protective immunity against virus reactivation from sensory neurons and reducing recurrent herpes infection and disease. However, in line with overall discussion, a successful HSV PPK therapeutic vaccine should incorporate (i) the T cell-attracting chemokines like CXCL9/10/11 to “pull” large numbers of antiviral functional tissue-resident CD4^+^ and CD8^+^ T_RM_ cells from the circulation into the sensory neurons and mucosal sites of HSV-infected animals and (ii) retention and expansion cytokines IL-7/IL-15 (IL-7R/IL-15R ligands) and chemokines CXCL17/CCL28 that are expected to “keep” long-term CD4^+^ and CD8^+^ T_RM_ cells within latently infected ganglions.

Prime/Pull/Keep (PPK) herpes vaccine strategies will have an unequivocally high medical impact by preventing recurrences and thus blinding recurrent herpetic disease because (i) they will achieve a needed breakthrough that failed in clinical trials over last 25 years by using a novel tissue-targeted PPK strategy to boost the number of protective HSV-specific CD8^+^ T_RM_ cells, in latently infected TG/DRG; (ii) they will selectively increase the number of functional CD8^+^ T_RM_ cells and retain them over the long term in latently infected TG/DRG; and (iii) they will fundamentally modify existing concepts of herpes immunity. Regardless of whether the proposed experiments lead to a clinical herpes immunotherapeutic, they will produce a new body of information regarding the poorly understood role of antiviral DRG/TG-resident CD8^+^ T cells in the protection against HSV reactivation.

Other critical barriers remain for developing a safe and efficient herpes simplex vaccine, including a limited understanding of the precise immune responses required for durable protection and viral control. Understanding how HSV evades the immune system is crucial, as studying the complex interactions between the virus and the host can help guide the development of vaccines that trigger strong protective immune responses. Developing effective vaccines or strategies largely depends on identifying the key immune correlates, which will guide the design of candidates that elicit the right quality and magnitude of immune responses, as well as strategies that recruit these immune cells to combat viral latency, immune evasion, and reactivation.

## Figures and Tables

**Table 1:** Herpes Vaccine Strategies—Mechanisms and Key Features.

Serial Number	Vaccine Approach	Mechanism of Action	Features
1.	Live-Attenuated Virus	Genetically weakened HSV replicates minimally to induce broad humoral and cellular immunity	Strong immunogenicity; risk of reactivation or safety concerns in immunocompromised
2.	Replication-Defective Virus	HSV strains deleted for essential replication genes, express viral proteins without producing virions	Induces T cell and antibody responses; safer than live-attenuated
3.	Protein Subunit Vaccine	Uses recombinant HSV glycoproteins (e.g., gD2) to induce neutralizing antibody responses	Safe; requires strong adjuvants; limited T cell activation
4.	Viral Vector Vaccine	Uses recombinant viruses (e.g., adenovirus, MVA) to deliver HSV genes to host cells	Potent T cell responses; pre-existing vector immunity can affect efficacy
5.	DNA Vaccine	Plasmid DNA encodes HSV antigens; delivered via electroporation or injection	Stable, easy to manufacture; moderate immunogenicity without adjuvants
6.	mRNA Vaccine	Delivers mRNA encoding HSV antigens via lipid nanoparticles; host cells express viral proteins	Highly immunogenic; induces both arms of adaptive immunity

**Table 2:** Summary of selected/recent Herpes Clinical trials.

Antigen(s), (Maker)	Format	Adjuvant	Route(s)	Endpoint(s) (Clinical)	Clinical Results Summary	Immunogenicity in HSV (+) Persons	Refs.
AGH1	mRNA (DNA)	>90% HLA diverse people have CD8 T cell responses to DNA versions. Targets HSV-1, HSV-2					
gB2/gC2/gD2/mICP0/tmICP4 (Moderna) mRNA		None	IM	Undisclosed			[141]
tgD2 (Chiron)	protein	Alum	IM	Rec	positive (weak)	↑ Ab, nAb	[111,112]
tgD2/tgB2 (Chiron)	protein	MF59	IM	Rec	Negative	↑ Ab, nAb	[112]
tgD2/ICP4 (Genocea)	protein	QS21	IM	Rec, Shed	positive	↑ Ab, nAb, ↑ “T cells”	[104]
32 HSV-2 peptides (Agenus)	peptides	QS21	IM	None	NA phase 1	↑ CD4, CD8 (weak)	[142]
HSV-2 UL5/29 del (Sanofi)	rincompvir	None	SC	None	NA phase 1	↑ CD4, CD8 (weak)	[106]
HSV-2 UL5/29 del (Sanofi)	rincompvir	None	SC	None	NA phase 1	↑ genital skin CD4 TRM	[106]
UL25, tUL 19, tgD2 (Sanofi)	protein	+GLA-SE	SCIM	None	NA phase 1	↑ Ab, nAb, CD4	
HSV-2 gH del (Cantab)	rincompvir	None	SC	Rec, Shed	negative	No immune data	[143]
HSV-2 ICP10del	repcompvir	?	?	“Recruitment” per web site 2025. Uneven FDA history			[144]
Undisclosed (GSK)	protein	MPL/QS21	IM	undisclosed	negative	Undisclosed	internet
gD2+targeting tag (Coridon)	DNA	None	IM	Rec	negative	↑ Ab, CD4 (weak)	[122]
gD2/UL46 (Vical)	DNA	Lipid	IM	undisclosed	negative	undisclosed	silence
DNA fragment(s) (Powdermed)	DNA via “gene gun”		Skin	Composition(s), clinical/immunogenicity data undisclosed			silence
gD2 (Apollon)	DNA	None	SC	NA	NA Phase I	No Ab or T cell boost	[145]

Abbreviations: NA = not applicable, m = mutated, g = glycoprotein, ICP = infected cell protein, del = deleted for HSV-2 replication/virulence genes, pro = protein, rincompvir = replication incompetent HSV-2, repcompvir = replication competent HSV-2, IM = intramuscular, SC = subcutaneous, Rec = recurrence. Shed = Anogenital or ocular HSV shedding. Recurrences = anogenital HSV shedding by daily PCR. The gene gun is a gold microparticle-mediated skin delivery device. Ab summarizes binding serum Ig, usually IgG. NAb = neutralizing antibody, usually serum. CD4, CD8 = PBMC CD4, CD8 responses to relevant antigen, ? = Unknown, Internet = press release. Silence = trial results not publicly reported.

## Abbreviations

PPK	Prime/Pull/Keep
HSV	Herpes Simplex Virus
HSV-1	Herpes Simplex Virus Type 1
HSV-2	Herpes Simplex Virus Type 2
ASYMP	Asymptomatic
SYMP	Symptomatic
TG	Trigeminal Ganglia
DRG	Dorsal Root Ganglia
VMC	Vaginal Mucocutaneous Tissue
TCR	T Cell Receptor
HLA	Human Leukocyte Antigen
APCs	Antigen-Presenting Cells
CALT	Conjunctival-Associated Lymphoid Tissue
gD	Glycoprotein D
T_RM_	Tissue-Resident Memory T Cell
T_EM_	Effector Memory T Cell
Tg	Transgenic
AV	Adenoviral Vectors
LNP	Lipid Nanoparticle
mRNA	Messenger Ribonucleic Acid
saRNA	Self-Amplifying RNA
PP	Prime/Pull
IEDB	Immune Epitope Database
WHO	World Health Organization

## References

[1] World Health Organization (2024). Herpes Simplex Virus.

[2] Harfouche M., AlMukdad S., Alareeki A., Osman A.M.M., Gottlieb S., Rowley J., Abu-Raddad L.J., Looker K.J. (2024). Estimated global and regional incidence and prevalence of herpes simplex virus infections and genital ulcer disease in 2020: Mathematical modelling analyses. Sex. Transm. Infect..

[3] Sanchez P.J., White N.O., Graf R.J., Taveras J. (2025). Management of neonates born to mothers with active genital herpes simplex virus infection: An alternative approach. Curr. Opin. Infect. Dis..

[4] Ramchandani M., Kong M., Tronstein E., Selke S., Mikhaylova A., Magaret A., Huang M.L., Johnston C., Corey L., Wald A. (2016). Herpes Simplex Virus Type 1 Shedding in Tears and Nasal and Oral Mucosa of Healthy Adults. Sex. Transm. Dis..

[5] Dasgupta G., BenMohamed L. (2011). Of mice and not humans: How reliable are animal models for evaluation of herpes CD8^+^-T cell-epitopes-based immunotherapeutic vaccine candidates?. Vaccine.

[6] Kuo T., Wang C., Badakhshan T., Chilukuri S., BenMohamed L. (2014). The challenges and opportunities for the development of a T-cell epitope-based herpes simplex vaccine. Vaccine.

[7] Belshe R.B., Leone P.A., Bernstein D.I., Wald A., Levin M.J., Stapleton J.T., Gorfinkel I., Morrow R.L.A., Ewell M.G., Stokes-Riner A. (2012). Efficacy Results of a Trial of a Herpes Simplex Vaccine. N. Engl. J. Med..

[8] Quadiri A., Prakash S., Vahed H., Tadros J.M., Sun M., Hormi-Carver K.K., Patel S.J., BenMohamed L. (2025). Therapeutic mucosal vaccination of herpes simplex virus type 2 infected guinea pigs with an adenovirus-based vaccine expressing the ribonucleotide reductase 2 and glycoprotein D induces local tissue-resident CD4^+^ and CD8^+^ TRM cells associated with protection against recurrent genital herpes. Front. Immunol..

[9] Meller N. (2025). Genital Herpes Simplex Virus Infections in Women-A Clinical Update. Clin. Obstet. Gynecol..

[10] Ashley R.L., Wald A. (1999). Genital herpes: Review of the epidemic and potential use of type-specific serology. Clin. Microbiol. Rev..

[11] Looker K.J., Elmes J.A.R., Gottlieb S.L., Schiffer J.T., Vickerman P., Turner K.M.E., Boily M.C. (2017). Effect of HSV-2 infection on subsequent HIV acquisition: An updated systematic review and meta-analysis. Lancet Infect. Dis..

[12] Gopinath D., Koe K.H., Maharajan M.K., Panda S. (2023). A Comprehensive Overview of Epidemiology, Pathogenesis and the Management of Herpes Labialis. Viruses.

[13] Chentoufi A.A., Dhanushkodi N.R., Srivastava R., Prakash S., Coulon P.A., Zayou L., Vahed H., Chentoufi H.A., Hormi-Carver K.K., BenMohamed L. (2022). Combinatorial Herpes Simplex Vaccine Strategies: From Bedside to Bench and Back. Front. Immunol..

[14] Koutsky L.A., Stevens C.E., Holmes K.K., Ashley R.L., Kiviat N.B., Critchlow C.W., Corey L. (1992). Underdiagnosis of genital herpes by current clinical and viral-isolation procedures. N. Engl. J. Med..

[15] Reeves W.C., Corey L., Adams H.G., Vontver L.A., Holmes K.K. (1981). Risk of recurrence after first episodes of genital herpes. Relation to HSV type and antibody response. N. Engl. J. Med..

[16] Stanberry L., Cunningham A., Mertz G., Mindel A., Peters B., Reitano M., Sacks S., Wald A., Wassilew S., Woolley P. (1999). New developments in the epidemiology, natural history and management of genital herpes. Antivir. Res..

[17] Chentoufi A.A., Kritzer E., Yu D.M., Nesburn A.B., Benmohamed L. (2012). Towards a rational design of an asymptomatic clinical herpes vaccine: The old, the new, and the unknown. Clin. Dev. Immunol..

[18] Cohen J.I. (2024). Therapeutic vaccines for herpesviruses. J. Clin. Investig..

[19] Cohen J.I. (2020). Herpesvirus latency. J. Clin. Investig..

[20] Melchjorsen J., Matikainen S., Paludan S.R. (2009). Activation and evasion of innate antiviral immunity by herpes simplex virus. Viruses.

[21] Awasthi S., Belshe R.B., Friedman H.M. (2014). Better neutralization of herpes simplex virus type 1 (HSV-1) than HSV-2 by antibody from recipients of GlaxoSmithKline HSV-2 glycoprotein D2 subunit vaccine. J. Infect. Dis..

[22] Knipe D.M., Corey L., Cohen J.I., Deal C.D. (2014). Summary and recommendations from a National Institute of Allergy and Infectious Diseases (NIAID) workshop on “Next Generation Herpes Simplex Virus Vaccines”. Vaccine.

[23] Dasgupta G., Chentoufi A.A., You S., Falatoonzadeh P., Urbano L.A., Akhtarmalik A., Nguyen K., Ablabutyan L., Nesburn A.B., BenMohamed L. (2011). Engagement of TLR2 reverses the suppressor function of conjunctiva CD4^+^CD25^+^ regulatory T cells and promotes herpes simplex virus epitope-specific CD4^+^CD25^−^ effector T cell responses. Investig. Ophthalmol. Vis. Sci..

[24] Verzosa A.L., McGeever L.A., Bhark S.J., Delgado T., Salazar N., Sanchez E.L. (2021). Herpes Simplex Virus 1 Infection of Neuronal and Non-Neuronal Cells Elicits Specific Innate Immune Responses and Immune Evasion Mechanisms. Front. Immunol..

[25] Allen S.J., Mott K.R., Chentoufi A.A., BenMohamed L., Wechsler S.L., Ballantyne C.M., Ghiasi H. (2011). CD11c controls herpes simplex virus 1 responses to limit virus replication during primary infection. J. Virol..

[26] Kollias C.M., Huneke R.B., Wigdahl B., Jennings S.R. (2015). Animal models of herpes simplex virus immunity and pathogenesis. J. Neurovirol.

[27] Hussain M.T., Stanfield B.A., Bernstein D.I. (2024). Small Animal Models to Study Herpes Simplex Virus Infections. Viruses.

[28] Truong N.R., Smith J.B., Sandgren K.J., Cunningham A.L. (2019). Mechanisms of Immune Control of Mucosal HSV Infection: A Guide to Rational Vaccine Design. Front. Immunol..

[29] Stern L., Emanuel Z., Traves R., Willis K., Purohit S.K., Samer C., Mak J.Y.W., Fairlie D.P., Tscharke D.C., Corbett A.J. (2025). Herpes simplex virus type 1 impairs mucosal-associated invariant T cells. mBio.

[30] Roy M., Lebeau L., Chessa C., Damour A., Ladram A., Oury B., Boutolleau D., Bodet C., Leveque N. (2019). Comparison of Anti-Viral Activity of Frog Skin Anti-Microbial Peptides Temporin-Sha and [K(3)]SHa to LL-37 and Temporin-Tb against Herpes Simplex Virus Type 1. Viruses.

[31] Vilas Boas L.C., de Lima L.M., Migliolo L., Mendes G.D., de Jesus M.G., Franco O.L., Silva P.A. (2017). Linear antimicrobial peptides with activity against herpes simplex virus 1 and Aichi virus. Biopolymers.

[32] Sanders L.S., Comar C.E., Srinivas K.P., Lalli J., Salnikov M., Lengyel J., Southern P., Mohr I., Wilson A.C., Rice S.A. (2023). Herpes Simplex Virus-1 ICP27 Nuclear Export Signal Mutants Exhibit Cell Type-Dependent Deficits in Replication and ICP4 Expression. J. Virol..

[33] Koffa M.D., Clements J.B., Izaurralde E., Wadd S., Wilson S.A., Mattaj I.W., Kuersten S. (2023). Herpes simplex virus ICP27 protein provides viral mRNAs with access to the cellular mRNA export pathway. EMBO J..

[34] Ostler J.B., Jones C. (2021). Stress Induced Transcription Factors Transactivate the Herpes Simplex Virus 1 Infected Cell Protein 27 (ICP27) Transcriptional Enhancer. Viruses.

[35] Birkenheuer C.H., Baines J.D. (2024). Aberrant RNA polymerase initiation and processivity on the genome of a herpes simplex virus 1 mutant lacking ICP27. J. Virol..

[36] Tormanen K., Matundan H.H., Wang S., Jaggi U., Mott K.R., Ghiasi H. (2022). Small Noncoding RNA (sncRNA1) within the Latency-Associated Transcript Modulates Herpes Simplex Virus 1 Virulence and the Host Immune Response during Acute but Not Latent Infection. J. Virol..

[37] Washington S.D., Edenfield S.I., Lieux C., Watson Z.L., Taasan S.M., Dhummakupt A., Bloom D.C., Neumann D.M. (2018). Depletion of the Insulator Protein CTCF Results in Herpes Simplex Virus 1 Reactivation In Vivo. J. Virol..

[38] Lucinda N., Figueiredo M.M., Pessoa N.L., Santos B.S., Lima G.K., Freitas A.M., Machado A.M., Kroon E.G., Antonelli L.R., Campos M.A. (2017). Dendritic cells, macrophages, NK and CD8^+^ T lymphocytes play pivotal roles in controlling HSV-1 in the trigeminal ganglia by producing IL1-beta, iNOS and granzyme B. Virol. J..

[39] Reinert L.S., Rashidi A.S., Tran D.N., Katzilieris-Petras G., Hvidt A.K., Gohr M., Fruhwurth S., Bodda C., Thomsen M.K., Vendelbo M.H. (2021). Brain immune cells undergo cGAS/STING-dependent apoptosis during herpes simplex virus type 1 infection to limit type I IFN production. J. Clin. Investig..

[40] Vollstedt S., Arnold S., Schwerdel C., Franchini M., Alber G., Di Santo J.P., Ackermann M., Suter M. (2004). Interplay between alpha/beta and gamma interferons with B, T, and natural killer cells in the defense against herpes simplex virus type 1. J. Virol..

[41] Leib D.A., Harrison T.E., Laslo K.M., Machalek M.A., Moorman N.J., Virgin H.W. (1999). Interferons regulate the phenotype of wild-type and mutant herpes simplex viruses in vivo. J. Exp. Med..

[42] Antony F., Pundkar C., Sandey M., Jaiswal A.K., Mishra A., Kumar A., Channappanavar R., Suryawanshi A. (2021). IFN-lambda Regulates Neutrophil Biology to Suppress Inflammation in Herpes Simplex Virus-1-Induced Corneal Immunopathology. J. Immunol..

[43] St Leger A.J., Koelle D.M., Kinchington P.R., Verjans G. (2021). Local Immune Control of Latent Herpes Simplex Virus Type 1 in Ganglia of Mice and Man. Front. Immunol..

[44] Ford E.S., Sholukh A.M., Boytz R., Carmack S.S., Klock A., Phasouk K., Shao D., Rossenkhan R., Edlefsen P.T., Peng T. (2021). B cells, antibody-secreting cells, and virus-specific antibodies respond to herpes simplex virus 2 reactivation in skin. J. Clin. Investig..

[45] Quadiri A., Kori L., Singh S.K., Anvikar A.R. (2022). Antibody Responses Against Plasmodium falciparum MSP3 Protein During Natural Malaria Infection in Individuals Living in Malaria-Endemic Regions of India. Proc. Natl. Acad. Sci. India Sect. B Biol. Sci..

[46] Laing K.J., Dong L., Sidney J., Sette A., Koelle D.M. (2012). Immunology in the Clinic Review Series; focus on host responses: T cell responses to herpes simplex viruses. Clin. Exp. Immunol..

[47] O’Neil T.R., Hu K., Truong N.R., Arshad S., Shacklett B.L., Cunningham A.L., Nasr N. (2021). The Role of Tissue Resident Memory CD4 T Cells in Herpes Simplex Viral and HIV Infection. Viruses.

[48] Posavad C.M., Remington M., Mueller D.E., Zhao L., Magaret A.S., Wald A., Corey L. (2010). Detailed characterization of T cell responses to herpes simplex virus-2 in immune seronegative persons. J. Immunol..

[49] Zhu J., Peng T., Johnston C., Phasouk K., Kask A.S., Klock A., Jin L., Diem K., Koelle D.M., Wald A. (2013). Immune surveillance by CD8alphaalpha^+^ skin-resident T cells in human herpes virus infection. Nature.

[50] Chentoufi A.A., Khan A.A., Srivastava R., Karan S., Lekbach Y., Vahed H., BenMohamed L. (2025). Dysfunctional Senescent Herpes Simplex Virus-Specific CD57^+^CD8^+^ T Cells Are Associated with Symptomatic Recurrent Ocular Herpes in Humans. Viruses.

[51] Liu J., Li Z. (2021). Resident Innate Immune Cells in the Cornea. Front. Immunol..

[52] Milligan G.N., Dudley-McClain K.L., Young C.G., Chu C.F. (2004). T-cell-mediated mechanisms involved in resolution of genital herpes simplex virus type 2 (HSV-2) infection of mice. J. Reprod. Immunol..

[53] Johnson A.J., Chu C.F., Milligan G.N. (2008). Effector CD4^+^ T-cell involvement in clearance of infectious herpes simplex virus type 1 from sensory ganglia and spinal cords. J. Virol..

[54] Lobo A.M., Agelidis A.M., Shukla D. (2019). Pathogenesis of herpes simplex keratitis: The host cell response and ocular surface sequelae to infection and inflammation. Ocul. Surf..

[55] Egan K., Hook L.M., LaTourette P., Desmond A., Awasthi S., Friedman H.M. (2020). Vaccines to prevent genital herpes. Transl. Res..

[56] Bernstein D.I. (2020). Use of the Guinea pig model of genital herpes to evaluate vaccines and antivirals: Review. Antivir. Res..

[57] Estes J.D., Wong S.W., Brenchley J.M. (2018). Nonhuman primate models of human viral infections. Nat. Rev. Immunol..

[58] Aravantinou M., Mizenina O., Calenda G., Kenney J., Frank I., Lifson J.D., Szpara M., Jing L., Koelle D.M., Teleshova N. (2017). Experimental Oral Herpes Simplex Virus-1 (HSV-1) Co-infection in Simian Immunodeficiency Virus (SIV)-Infected Rhesus Macaques. Front. Microbiol..

[59] Wang K., Jordan T., Dowdell K., Herbert R., Moore I.N., Koelle D.M., Cohen J.I. (2024). A nonhuman primate model for genital herpes simplex virus 2 infection that results in vaginal vesicular lesions, virus shedding, and seroconversion. PLoS Pathog..

[60] Jia Z., Zhang D., Zhu L., Xue J. (2025). Animal models of human herpesvirus infection. Anim. Model. Exp. Med..

[61] Svensson A., Bellner L., Magnusson M., Eriksson K. (2007). Role of IFN-alpha/beta signaling in the prevention of genital herpes virus type 2 infection. J. Reprod. Immunol..

[62] Stuart P.M., Keadle T.L. (2012). Recurrent herpetic stromal keratitis in mice: A model for studying human HSK. Clin. Dev. Immunol..

[63] Chentoufi A.A., Dasgupta G., Christensen N.D., Hu J., Choudhury Z.S., Azeem A., Jester J.V., Nesburn A.B., Wechsler S.L., BenMohamed L. (2010). A novel HLA (HLA-A*0201) transgenic rabbit model for preclinical evaluation of human CD8^+^ T cell epitope-based vaccines against ocular herpes. J. Immunol..

[64] BenMohamed L., Krishnan R., Longmate J., Auge C., Low L., Primus J., Diamond D.J. (2000). Induction of CTL response by a minimal epitope vaccine in HLA A*0201/DR1 transgenic mice: Dependence on HLA class II restricted T(H) response. Hum. Immunol..

[65] Srivastava R., Khan A.A., Spencer D., Vahed H., Lopes P.P., Thai N.T., Wang C., Pham T.T., Huang J., Scarfone V.M. (2015). HLA-A02:01-restricted epitopes identified from the herpes simplex virus tegument protein VP11/12 preferentially recall polyfunctional effector memory CD8^+^ T cells from seropositive asymptomatic individuals and protect humanized HLA-A*02:01 transgenic mice against ocular herpes. J. Immunol..

[66] Chentoufi A.A., Zhang X., Lamberth K., Dasgupta G., Bettahi I., Nguyen A., Wu M., Zhu X., Mohebbi A., Buus S. (2008). HLA-A*0201-restricted CD8^+^ cytotoxic T lymphocyte epitopes identified from herpes simplex virus glycoprotein D. J. Immunol..

[67] Dervillez X., Qureshi H., Chentoufi A.A., Khan A.A., Kritzer E., Yu D.C., Diaz O.R., Gottimukkala C., Kalantari M., Villacres M.C. (2013). Asymptomatic HLA-A*02:01-restricted epitopes from herpes simplex virus glycoprotein B preferentially recall polyfunctional CD8^+^ T cells from seropositive asymptomatic individuals and protect HLA transgenic mice against ocular herpes. J. Immunol..

[68] Chentoufi A.A., Binder N.R., Berka N., Durand G., Nguyen A., Bettahi I., Maillere B., BenMohamed L. (2008). Asymptomatic human CD4^+^ cytotoxic T-cell epitopes identified from herpes simplex virus glycoprotein B. J. Virol..

[69] Zhang X., Castelli F.A., Zhu X., Wu M., Maillere B., BenMohamed L. (2008). Gender-dependent HLA-DR-restricted epitopes identified from herpes simplex virus type 1 glycoprotein D. Clin. Vaccine Immunol. CVI.

[70] Khan A.A., Srivastava R., Chentoufi A.A., Kritzer E., Chilukuri S., Garg S., Yu D.C., Vahed H., Huang L., Syed S.A. (2017). Bolstering the Number and Function of HSV-1-Specific CD8^+^ Effector Memory T Cells and Tissue-Resident Memory T Cells in Latently Infected Trigeminal Ganglia Reduces Recurrent Ocular Herpes Infection and Disease. J. Immunol..

[71] Chentoufi A.A., Prakash S., Vahed H., Karan S., Quadiri A., Nesburn A.B., BenMohamed L. (2025). A Tissue-Targeted Prime/Pull/Keep Therapeutic Herpes Simplex Virus Vaccine Protect Against Recurrent Ocular Herpes Infection and Disease in HLA-A*0201 Transgenic Rabbits. J. Virol..

[72] Roy S., Coulon P.G., Prakash S., Srivastava R., Geertsema R., Dhanushkodi N., Lam C., Nguyen V., Gorospe E., Nguyen A.M. (2019). Blockade of PD-1 and LAG-3 Immune Checkpoints Combined with Vaccination Restores the Function of Antiviral Tissue-Resident CD8^+^ T_RM_ Cells and Reduces Ocular Herpes Simplex Infection and Disease in HLA Transgenic Rabbits. J. Virol..

[73] Khan A.A., Srivastava R., Vahed H., Roy S., Walia S.S., Kim G.J., Fouladi M.A., Yamada T., Ly V.T., Lam C. (2018). Human Asymptomatic Epitope Peptide/CXCL10-Based Prime/Pull Vaccine Induces Herpes Simplex Virus-Specific Gamma Interferon-Positive CD107^+^ CD8^+^ T Cells That Infiltrate the Corneas and Trigeminal Ganglia of Humanized HLA Transgenic Rabbits and Protect against Ocular Herpes Challenge. J. Virol..

[74] Srivastava R., Khan A.A., Huang J., Nesburn A.B., Wechsler S.L., BenMohamed L. (2015). A Herpes Simplex Virus Type 1 Human Asymptomatic CD8^+^ T-Cell Epitopes-Based Vaccine Protects Against Ocular Herpes in a “Humanized” HLA Transgenic Rabbit Model. Investig. Ophthalmol. Vis. Sci..

[75] Berman E.J., Hill J.M. (1985). Spontaneous ocular shedding of HSV-1 in latently infected rabbits. Investig. Ophthalmol. Vis. Sci..

[76] Perng G.C., Maguen B., Jin L., Mott K.R., Kurylo J., BenMohamed L. (2002). A novel herpes simplex virus type 1 transcript (AL-RNA) antisense to the 5’ end of the latency-associated transcript produces a protein in infected rabbits. J. Virol..

[77] Prakash S., Roy S., Srivastava R., Coulon P.G., Dhanushkodi N.R., Vahed H., Jankeel A., Geertsema R., Amezquita C., Nguyen L. (2020). Unique molecular signatures of antiviral memory CD8^+^ T cells associated with asymptomatic recurrent ocular herpes. Sci. Rep..

[78] Esteves P.J., Abrantes J., Baldauf H.M., BenMohamed L., Chen Y., Christensen N., Gonzalez-Gallego J., Giacani L., Hu J., Kaplan G. (2018). The wide utility of rabbits as models of human diseases. Exp. Mol. Med..

[79] Mackay L.K., Minnich M., Kragten N.A., Liao Y., Nota B., Seillet C. (2016). Hobit and Blimp1 instruct a universal transcriptional program of tissue residency in lymphocytes. Science.

[80] Khan A.A., Srivastava R., Chentoufi A.A., Geertsema R., Thai N.T., Dasgupta G., Osorio N., Kalantari M., Nesburn A.B., Wechsler S.L. (2015). Therapeutic immunization with a mixture of herpes simplex virus 1 glycoprotein D-derived “asymptomatic” human CD8^+^ T-cell epitopes decreases spontaneous ocular shedding in latently infected HLA transgenic rabbits: Association with low frequency of local PD-1^+^ TIM-3^+^ CD8^+^ exhausted T cells. J. Virol..

[81] Perng G.C., Osorio N., Jiang X., Geertsema R., Hsiang C., Brown D. (2015). Large Amounts of Reactivated Virus in Tears Precedes Recurrent Herpes Stromal Keratitis in Stressed Rabbits Latently Infected with Herpes Simplex Virus. Curr Eye Res..

[82] Bourne N., Perry C.L., Banasik B.N., Miller A.L., White M., Pyles R.B., Schafer H., Milligan G.N. (2019). Increased Frequency of Virus Shedding by Herpes Simplex Virus 2-Infected Guinea Pigs in the Absence of CD4^+^ T Lymphocytes. J. Virol..

[83] Bourne N., Banasik B.N., Perry C.L., Miller A.L., White M., Pyles R.B., Milligan G.N. (2019). Development of disease and immunity at the genital epithelium following intrarectal inoculation of male guinea pigs with herpes simplex virus type 2. Virology.

[84] Hook L.M., Friedman H.M., Awasthi S. (2021). Guinea Pig and Mouse Models for Genital Herpes Infection. Curr. Protoc..

[85] Awasthi S., Lubinski J.M., Shaw C.E., Barrett S.M., Cai M., Wang F., Betts M., Kingsley S., Distefano D.J., Balliet J.W. (2011). Immunization with a vaccine combining herpes simplex virus 2 (HSV-2) glycoprotein C (gC) and gD subunits improves the protection of dorsal root ganglia in mice and reduces the frequency of recurrent vaginal shedding of HSV-2 DNA in guinea pigs compared to immunization with gD alone. J. Virol..

[86] Awasthi S., Balliet J.W., Flynn J.A., Lubinski J.M., Shaw C.E., DiStefano D.J., Cai M., Brown M., Smith J.F., Kowalski R. (2014). Protection provided by a herpes simplex virus 2 (HSV-2) glycoprotein C and D subunit antigen vaccine against genital HSV-2 infection in HSV-1-seropositive guinea pigs. J. Virol..

[87] Hook L.M., Cairns T.M., Awasthi S., Brooks B.D., Ditto N.T., Eisenberg R.J., Cohen G.H., Friedman H.M. (2018). Vaccine-induced antibodies to herpes simplex virus glycoprotein D epitopes involved in virus entry and cell-to-cell spread correlate with protection against genital disease in guinea pigs. PLoS Pathog..

[88] Egan K., Hook L.M., Naughton A., Friedman H.M., Awasthi S. (2020). Herpes simplex virus type 2 trivalent protein vaccine containing glycoproteins C, D and E protects guinea pigs against HSV-1 genital infection. Hum. Vaccin. Immunother..

[89] Chentoufi A.A., Kritzer E., Tran M.V., Dasgupta G., Lim C.H., Yu D.C. (2011). The herpes simplex virus 1 latency-associated transcript promotes functional exhaustion of virus-specific CD8+ T cells in latently infected trigeminal ganglia: A novel immune evasion mechanism. J. Virol..

[90] Chentoufi A.A., Dervillez X., Dasgupta G., Nguyen C., Kabbara W.K., Jiang X. (2012). The Herpes Simplex Virus Type 1 Latency Associated Transcript Inhibits Phenotypic and Functional Maturation of Dendritic Cells. Viral Immunol..

[91] Bernstein D.I., Cardin R.D., Bravo F.J., Awasthi S., Lu P., Pullum D.A. (2019). Successful application of prime and pull strategy for a therapeutic HSV vaccine. NPJ Vaccines.

[92] Srivastava R., Roy S., Coulon P.G., Vahed H., Prakash S., Dhanushkodi N. (2019). Therapeutic Mucosal Vaccination of Herpes Simplex Virus 2-Infected Guinea Pigs with Ribonucleotide Reductase 2 (RR2) Protein Boosts Antiviral Neutralizing Antibodies and Local Tissue-Resident CD4(+) and CD8(+) TRM Cells Associated with Protection against Recurrent Genital Herpes. J. Virol..

[93] Bernstein D.I., Pullum D.A., Cardin R.D., Bravo F.J., Dixon D.A., Kousoulas K.G. (2019). The HSV-1 live attenuated VC2 vaccine provides protection against HSV-2 genital infection in the guinea pig model of genital herpes. Vaccine.

[94] Mott K.R., Chentoufi A.A., Carpenter D., BenMohamed L., Wechsler S., Ghiasi H. (2009). A glycoprotein K (gK) CD8^+^ T-cell epitope of herpes simplex virus types 1 and 2 increases ocular virus replication and pathogenicity. Investig. Ophthalmol. Vis. Sci..

[95] Taneja V., David C.S. (1999). HLA class II transgenic mice as models of human diseases. Immunol Rev..

[96] Quadiri A., Prakash S., Dhanushkodi N.R., Singer M., Zayou L., Shaik A.M., Sun M., Suzer B., Lau L.S.L., Chilukurri A. (2024). Therapeutic prime/pull vaccination of HSV-2-infected guinea pigs with the ribonucleotide reductase 2 (RR2) protein and CXCL11 chemokine boosts antiviral local tissue-resident and effector memory CD4^+^ and CD8^+^ T cells and protects against recurrent genital herpes. J. Virol..

[97] Fan Y., Huang Z.Y., Cao C.C., Chen C.S., Chen Y.X., Fan D.D., He J., Hou H.L., Hu L., Hu X.T. (2013). Genome of the Chinese tree shrew. Nat. Commun..

[98] Darai G., Schwaier A., Komitowski D., Munk K. (1978). Experimental infection of Tupaia belangeri (tree shrews) with herpes simplex virus types 1 and 2. J. Infect. Dis..

[99] Stanfield B.A., Kousoulas K.G., Fernandez A., Gershburg E. (2021). Rational Design of Live-Attenuated Vaccines against Herpes Simplex Viruses. Viruses.

[100] Mori I., Liu B., Goshima F., Ito H., Koide N., Yoshida T., Yokochi T., Kimura Y., Nishiyama Y. (2005). HF10, an attenuated herpes simplex virus (HSV) type 1 clone, lacks neuroinvasiveness and protects mice against lethal challenge with HSV types 1 and 2. Microbes Infect..

[101] Joyce J.D., Patel A.K., Murphy B., Carr D.J.J., Gershburg E., Bertke A.S. (2021). Assessment of Two Novel Live-Attenuated Vaccine Candidates for Herpes Simplex Virus 2 (HSV-2) in Guinea Pigs. Vaccines.

[102] Bloom D.C., Feller J., McAnany P., Vilaboa N., Voellmy R. (2015). Replication-Competent Controlled Herpes Simplex Virus. J. Virol..

[103] Carr D.J.J., Berube A., Gershburg E. (2021). The Durability of Vaccine Efficacy against Ocular HSV-1 Infection Using ICP0 Mutants 0∆NLS and 0∆RING Is Lost over Time. Pathogens.

[104] Chang J.Y., Balch C., Oh H.S. (2024). Toward the Eradication of Herpes Simplex Virus: Vaccination and Beyond. Viruses.

[105] Royer D.J., Gurung H.R., Jinkins J.K., Geltz J.J., Wu J.L., Halford W.P., Carr D.J.J. (2016). A Highly Efficacious Herpes Simplex Virus 1 Vaccine Blocks Viral Pathogenesis and Prevents Corneal Immunopathology via Humoral Immunity. J. Virol..

[106] Dropulic L.K., Oestreich M.C., Pietz H.L., Laing K.J., Hunsberger S., Lumbard K., Garabedian D., Turk S.P., Chen A., Hornung R.L. (2019). A Randomized, Double-Blinded, Placebo-Controlled, Phase 1 Study of a Replication-Defective Herpes Simplex Virus (HSV) Type 2 Vaccine, HSV529, in Adults With or Without HSV Infection. J. Infect. Dis..

[107] Pati R., Shevtsov M., Sonawane A. (2018). Nanoparticle Vaccines Against Infectious Diseases. Front. Immunol..

[108] Quadiri A., Kalia I., Kashif M., Singh A.P. (2020). Identification and characterization of protective CD8^+^ T-epitopes in a malaria vaccine candidate SLTRiP. Immun. Inflamm. Dis..

[109] Awasthi S., Huang J., Shaw C., Friedman H.M. (2014). Blocking herpes simplex virus 2 glycoprotein E immune evasion as an approach to enhance efficacy of a trivalent subunit antigen vaccine for genital herpes. J. Virol..

[110] Bernstein D.I., Wald A., Warren T., Fife K., Tyring S., Lee P., Van Wagoner N., Magaret A., Flechtner J.B., Tasker S. (2017). Therapeutic Vaccine for Genital Herpes Simplex Virus-2 Infection: Findings From a Randomized Trial. J. Infect. Dis..

[111] Straus S.E., Corey L., Burke R.L., Savarese B., Barnum G., Krause P.R., Kost R.G., Meier J.L., Sekulovich R., Adair S.F. (1994). Placebo-controlled trial of vaccination with recombinant glycoprotein D of herpes simplex virus type 2 for immunotherapy of genital herpes. Lancet.

[112] Corey L., Langenberg A.G., Ashley R., Sekulovich R.E., Izu A.E., Douglas J.M., Handsfield H.H., Warren T., Marr L., Tyring S. (1999). Recombinant glycoprotein vaccine for the prevention of genital HSV-2 infection: Two randomized controlled trials. Chiron HSV Vaccine Study Group. JAMA.

[113] Skoberne M., Cardin R., Lee A., Kazimirova A., Zielinski V., Garvie D., Lundberg A., Larson S., Bravo F.J., Bernstein D.I. (2013). An adjuvanted herpes simplex virus 2 subunit vaccine elicits a T cell response in mice and is an effective therapeutic vaccine in Guinea pigs. J. Virol..

[114] Van Wagoner N., Fife K., Leone P.A., Bernstein D.I., Warren T., Panther L., Novak R.M., Beigi R., Kriesel J., Tyring S. (2018). Effects of Different Doses of GEN-003, a Therapeutic Vaccine for Genital Herpes Simplex Virus-2, on Viral Shedding and Lesions: Results of a Randomized Placebo-Controlled Trial. J. Infect. Dis..

[115] Bernstein D.I., Flechtner J.B., McNeil L.K., Heineman T., Oliphant T., Tasker S., Wald A., Hetherington S., Genocea Study Group (2019). Therapeutic HSV-2 vaccine decreases recurrent virus shedding and recurrent genital herpes disease. Vaccine.

[116] Mo A., Musselli C., Chen H., Pappas J., Leclair K., Liu A., Chicz R.M., Truneh A., Monks S., Levey D.L. (2011). A heat shock protein based polyvalent vaccine targeting HSV-2: CD4^+^ and CD8^+^ cellular immunity and protective efficacy. Vaccine.

[117] Hamley I.W. (2022). Peptides for Vaccine Development. ACS Appl. Bio Mater..

[118] Kaufmann J.K., Flechtner J.B. (2016). Evolution of rational vaccine designs for genital herpes immunotherapy. Curr. Opin. Virol..

[119] Minaya M.A., Korom M., Wang H., Belshe R.B., Morrison L.A. (2017). The herpevac trial for women: Sequence analysis of glycoproteins from viruses obtained from infected subjects. PLoS ONE.

[120] Stanfield B.A., Bravo F.J., Dixon D.A., Chouljenko V.N., Kousoulas K.G., Bernstein D.I. (2022). Cross protective efficacy of the Non-Neurotropic live attenuated herpes simplex virus type 1 vaccine VC-2 is enhanced by intradermal vaccination and deletion of glycoprotein G. Vaccine.

[121] Veselenak R.L., Shlapobersky M., Pyles R.B., Wei Q., Sullivan S.M., Bourne N. (2012). A Vaxfectin((R))-adjuvanted HSV-2 plasmid DNA vaccine is effective for prophylactic and therapeutic use in the guinea pig model of genital herpes. Vaccine.

[122] Dutton J.L., Woo W.P., Chandra J., Xu Y., Li B., Finlayson N., Griffin P., Frazer I.H. (2016). An escalating dose study to assess the safety, tolerability and immunogenicity of a Herpes Simplex Virus DNA vaccine, COR-1. Hum. Vaccin. Immunother..

[123] Kim H.C., Oh D.S., Park J.H., Kim H.J., Seo Y.B., Yoo H.J., Jang H.S., Shin J., Kim C.W., Kwon M.S. (2020). Multivalent DNA vaccine protects against genital herpes by T-cell immune induction in vaginal mucosa. Antivir. Res..

[124] Us D. (2006). Herpes simplex virus vaccine studies: From past to present. Mikrobiyol. Bul..

[125] Cappel R. (1976). Comparison of the humoral and cellular immune response after immunization with live, UV inactivated herpes simplex virus and a subunit vaccine and efficacy of these immunizations. Arch. Virol..

[126] Metcalf J.F. (1980). Protection from experimental ocular herpetic keratitis by a heat-killed virus vaccine. Arch. Ophthalmol..

[127] Rajcani J., Kutinova L., Vonka V. (1980). Restriction of latent herpes virus infection in rabbits immunized with subviral herpes simplex virus vaccine. Acta Virol..

[128] Hoshino Y., Pesnicak L., Dowdell K.C., Lacayo J., Dudek T., Knipe D.M., Straus S.E., Cohen J.I. (2008). Comparison of immunogenicity and protective efficacy of genital herpes vaccine candidates herpes simplex virus 2 dl5-29 and dl5-29-41L in mice and guinea pigs. Vaccine.

[129] Liu X., Broberg E., Watanabe D., Dudek T., Deluca N., Knipe D.M. (2009). Genetic engineering of a modified herpes simplex virus 1 vaccine vector. Vaccine.

[130] Diaz F., Gregory S., Nakashima H., Viapiano M.S., Knipe D.M. (2018). Intramuscular delivery of replication-defective herpes simplex virus gives antigen expression in muscle syncytia and improved protection against pathogenic HSV-2 strains. Virology.

[131] Vagvala S.P., Thebeau L.G., Wilson S.R., Morrison L.A. (2009). Virus-encoded b7-2 costimulation molecules enhance the protective capacity of a replication-defective herpes simplex virus type 2 vaccine in immunocompetent mice. J. Virol..

[132] Stanfield B.A., Rider P.J.F., Caskey J., Del Piero F., Kousoulas K.G. (2018). Intramuscular vaccination of guinea pigs with the live-attenuated human herpes simplex vaccine VC2 stimulates a transcriptional profile of vaginal Th17 and regulatory Tr1 responses. Vaccine.

[133] Flechtner J.B., Long D., Larson S., Clemens V., Baccari A., Kien L., Chan J., Skoberne M., Brudner M., Hetherington S. (2016). Immune responses elicited by the GEN-003 candidate HSV-2 therapeutic vaccine in a randomized controlled dose-ranging phase 1/2a trial. Vaccine.

[134] Straus S.E., Wald A., Kost R.G., McKenzie R., Langenberg A.G., Hohman P., Lekstrom J., Cox E., Nakamura M., Sekulovich R. (1997). Immunotherapy of recurrent genital herpes with recombinant herpes simplex virus type 2 glycoproteins D and B: Results of a placebo-controlled vaccine trial. J. Infect. Dis..

[135] Stanberry L.R. (2004). Clinical trials of prophylactic and therapeutic herpes simplex virus vaccines. Herpes.

[136] Bromberg V., Hook L.M., Lubinski J.M., Syeda Z., Egan K.P., Cohen G.H., Awasthi S., Friedman H.M. (2025). Seroconversion Is Misleading as a Test for HSV-2 Infection in Prophylactic Genital Herpes Vaccine Trials: Results of Vaccine Studies in Guinea Pigs. Viruses.

[137] Egan K.P., Awasthi S., Tebaldi G., Hook L.M., Naughton A.M., Fowler B.T., Beattie M., Alameh M.G., Weissman D., Cohen G.H. (2023). A Trivalent HSV-2 gC2, gD2, gE2 Nucleoside-Modified mRNA-LNP Vaccine Provides Outstanding Protection in Mice against Genital and Non-Genital HSV-1 Infection, Comparable to the Same Antigens Derived from HSV-1. Viruses.

[138] Hook L.M., Awasthi S., Cairns T.M., Alameh M.G., Fowler B.T., Egan K.P., Sung M.M.H., Weissman D., Cohen G.H., Friedman H.M. (2022). Antibodies to Crucial Epitopes on HSV-2 Glycoprotein D as a Guide to Dosing an mRNA Genital Herpes Vaccine. Viruses.

[139] Awasthi S., Friedman H.M. (2022). An mRNA vaccine to prevent genital herpes. Transl. Res..

[140] Awasthi S., Knox J.J., Desmond A., Alameh M.G., Gaudette B.T., Lubinski J.M., Naughton A., Hook L.M., Egan K.P., Tam Y.K. (2021). Trivalent nucleoside-modified mRNA vaccine yields durable memory B cell protection against genital herpes in preclinical models. J. Clin. Investig..

[141] Awasthi S., Hook L.M., Pardi N., Wang F., Myles A., Cancro M.P., Cohen G.H., Weissman D., Friedman H.M. (2019). Nucleoside-modified mRNA encoding HSV-2 glycoproteins C, D, and E prevents clinical and subclinical genital herpes. Sci. Immunol..

[142] Wald A., Koelle D.M., Fife K., Warren T., Leclair K., Chicz R.M., Monks S., Levey D.L., Musselli C., Srivastava P.K. (2011). Safety and immunogenicity of long HSV-2 peptides complexed with rhHsc70 in HSV-2 seropositive persons. Vaccine.

[143] de Bruyn G., Vargas-Cortez M., Warren T., Tyring S.K., Fife K.H., Lalezari J. (2006). A randomized controlled trial of a replication defective (gH deletion) herpes simplex virus vaccine for the treatment of recurrent genital herpes among immunocompetent subjects. Vaccine.

[144] Casanova G., Cancela R., Alonzo L., Benuto R., Magana Mdel C., Hurley D.R. (2002). A double-blind study of the efficacy and safety of the ICP10deltaPK vaccine against recurrent genital HSV-2 infections. Cutis.

[145] Cattamanchi A., Posavad C.M., Wald A., Baine Y., Moses J., Higgins T.J. (2008). Phase I study of a herpes simplex virus type 2 (HSV-2) DNA vaccine administered to healthy, HSV-2-seronegative adults by a needle-free injection system. Clin Vaccine Immunol..

[146] Nidetz N.F., McGee M.C., Tse L.V., Li C., Cong L., Li Y. (2020). Adeno-associated viral vector-mediated immune responses: Understanding barriers to gene delivery. Pharmacol Ther..

[147] Kashif M., Quadiri A., Singh A.P. (2021). Essential role of a Plasmodium berghei heat shock protein (PBANKA_0938300) in gametocyte development. Sci. Rep..

[148] Halperin S.A., Ye L., MacKinnon-Cameron D., Smith B., Cahn P.E., Ruiz-Palacios G.M., Ikram A., Lanas F., Lourdes Guerrero M., Munoz Navarro S.R. (2022). Final efficacy analysis, interim safety analysis, and immunogenicity of a single dose of recombinant novel coronavirus vaccine (adenovirus type 5 vector) in adults 18 years and older: An international, multicentre, randomised, double-blinded, placebo-controlled phase 3 trial. Lancet.

[149] Wang S.Y., Liu W.Q., Li Y.Q., Li J.X., Zhu F.C. (2023). A China-developed adenovirus vector-based COVID-19 vaccine: Review of the development and application of Ad5-nCov. Expert. Rev. Vaccines.

[150] Madavaraju K., Koganti R., Volety I., Yadavalli T., Shukla D. (2020). Herpes Simplex Virus Cell Entry Mechanisms: An Update. Front. Cell Infect. Microbiol..

[151] Wan M., Yang X., Sun J., Ding X., Chen Z., Su W., Cai L., Hou A., Sun B., Gao F. (2023). An Adenovirus-Based Recombinant Herpes Simplex Virus 2 (HSV-2) Therapeutic Vaccine Is Highly Protective against Acute and Recurrent HSV-2 Disease in a Guinea Pig Model. Viruses.

[152] Aubert M., Haick A.K., Strongin D.E., Klouser L.M., Loprieno M.A., Stensland L., Santo T.K., Huang M.L., Hyrien O., Stone D. (2024). Gene editing for latent herpes simplex virus infection reduces viral load and shedding in vivo. Nat. Commun..

[153] Leong K.Y., Tham S.K., Poh C.L. (2025). Revolutionizing immunization: A comprehensive review of mRNA vaccine technology and applications. Virol. J..

[154] Quadiri A., Prakash S., Zayou L., Dhanushkodi N.R., Chilukuri A., Ryan G., Wang K., Vahed H., Chentoufi A.A., BenMohamed L. (2025). A Spike-Based mRNA Vaccine Encapsulated in Phospholipid 1,2-Dioleoyl-sn-Glycero-3-PhosphoEthanolamine Containing Lipid Nanoparticles Induced Potent B- and T-Cell Responses Associated with Protection Against SARS-CoV-2 Infection and COVID-19-like Symptoms in Hamsters. Vaccines.

[155] Vahed H., Prakash S., Quadiri A., Ibraim I.C., Omorogieva E., Patel S., Tadros J., Liao E.J., Lau L., Chentoufi A.A. (2025). A pan-beta-coronavirus vaccine bearing conserved and asymptomatic B- and T-cell epitopes protects against highly pathogenic Delta and highly transmissible Omicron SARS-CoV-2 variants. Hum. Vaccin. Immunother..

[156] Prakash S., Dhanushkodi N.R., Zayou L., Ibraim I.C., Quadiri A., Coulon P.G., Tifrea D.F., Suzer B., Shaik A.M., Chilukuri A. (2024). Cross-protection induced by highly conserved human B, CD4^+^, and CD8^+^ T-cell epitopes-based vaccine against severe infection, disease, and death caused by multiple SARS-CoV-2 variants of concern. Front. Immunol..

[157] Chaudhary N., Weissman D., Whitehead K.A. (2021). mRNA vaccines for infectious diseases: Principles, delivery and clinical translation. Nat. Rev. Drug Discov..

[158] Akingbola A., Adegbesan A., Adegoke K., Idahor C., Mariaria P., Peters F., Salami R.A., Ojo O., Nwaeze E., Abdullahi O. (2025). Comparing Moderna’s mRNA-1083 and Pfizer’s dual-target mRNA vaccines for influenza and COVID-19. NPJ Vaccines.

[159] Zayou L., Prakash S., Vahed H., Dhanushkodi N.R., Quadiri A., Belmouden A., Lemkhente Z., Chentoufi A., Gil D., Ulmer J.B. (2025). Dynamics of spike-specific neutralizing antibodies across five-year emerging SARS-CoV-2 variants of concern reveal conserved epitopes that protect against severe COVID-19. Front. Immunol..

[160] Dhanushkodi N.R., Prakash S., Quadiri A., Zayou L., Srivastava R., Shaik A.M., Suzer B., Ibraim I.C., Landucci G., Tifrea D.F. (2024). Antiviral and Anti-Inflammatory Therapeutic Effect of RAGE-Ig Protein against Multiple SARS-CoV-2 Variants of Concern Demonstrated in K18-hACE2 Mouse and Syrian Golden Hamster Models. J. Immunol..

[161] Pardi N., Hogan M.J., Porter F.W., Weissman D. (2018). mRNA vaccines-A new era in vaccinology. Nat. Rev. Drug Discov..

[162] Verbeke R., Hogan M.J., Lore K., Pardi N. (2022). Innate immune mechanisms of mRNA vaccines. Immunity.

[163] Egan K.P., Hook L.M., Naughton A., Pardi N., Awasthi S., Cohen G.H., Weissman D., Friedman H.M. (2020). An HSV-2 nucleoside-modified mRNA genital herpes vaccine containing glycoproteins gC, gD, and gE protects mice against HSV-1 genital lesions and latent infection. PLoS Pathog..

[164] Awasthi S., Hook L.M., Shaw C.E., Pahar B., Stagray J.A., Liu D., Veazey R.S., Friedman H.M. (2017). An HSV-2 Trivalent Vaccine Is Immunogenic in Rhesus Macaques and Highly Efficacious in Guinea Pigs. PLoS Pathog..

[165] Shin H., Iwasaki A. (2012). A vaccine strategy that protects against genital herpes by establishing local memory T cells. Nature.

[166] Tregoning J.S., Buffa V., Oszmiana A., Klein K., Walters A.A., Shattock R.J. (2013). A “prime-pull” vaccine strategy has a modest effect on local and systemic antibody responses to HIV gp140 in mice. PLoS ONE.

[167] Zayou L., Prakash S., Dhanushkodi N.R., Quadiri A., Ibraim I.C., Singer M., Salem A., Shaik A.M., Suzer B., Chilukuri A. (2023). A multi-epitope/CXCL11 prime/pull coronavirus mucosal vaccine boosts the frequency and the function of lung-resident memory CD4^+^ and CD8^+^ T cells and enhanced protection against COVID-19-like symptoms and death caused by SARS-CoV-2 infection. J. Virol..

[168] Gebhardt T., Whitney P.G., Zaid A., Mackay L.K., Brooks A.G., Heath W.R., Carbone F.R., Mueller S.N. (2011). Different patterns of peripheral migration by memory CD4^+^ and CD8^+^ T cells. Nature.

[169] Mackay L.K., Wakim L., van Vliet C.J., Jones C.M., Mueller S.N., Bannard O., Fearon D.T., Heath W.R., Carbone F.R. (2012). Maintenance of T cell function in the face of chronic antigen stimulation and repeated reactivation for a latent virus infection. J. Immunol..

[170] Mackay L.K., Stock A.T., Ma J.Z., Jones C.M., Kent S.J., Mueller S.N., Heath W.R., Carbone F.R., Gebhardt T. (2012). Long-lived epithelial immunity by tissue-resident memory T (TRM) cells in the absence of persisting local antigen presentation. Proc. Natl. Acad. Sci. USA.

[171] Masopust D., Picker L.J. (2012). Hidden memories: Frontline memory T cells and early pathogen interception. J. Immunol..

[172] Suni M.A., Ghanekar S.A., Houck D.W., Maecker H.T., Wormsley S.B., Picker L.J., Moss R.B., Maino V.C. (2001). CD4^+^CD8(dim) T lymphocytes exhibit enhanced cytokine expression, proliferation and cytotoxic activity in response to HCMV and HIV-1 antigens. Eur. J. Immunol..

[173] Jiang X., Chentoufi A.A., Hsiang C., Carpenter D., Osorio N., Benmohamed L., Fraser N.W., Jones C., Wechsler S.L. (2010). The herpes simplex virus type 1 latency associated transcript (LAT) can protect neuronal derived C1300 and Neuro2A cells from Granzyme B induced apoptosis and CD8 T-cell killing. J. Virol..

[174] Harari A., Enders F.B., Cellerai C., Bart P.A., Pantaleo G. (2009). Distinct profiles of cytotoxic granules in memory CD8 T cells correlate with function, differentiation stage, and antigen exposure. J. Virol..

[175] Jameson S.C., Masopust D. (2009). Diversity in T cell memory: An embarrassment of riches. Immunity.

[176] Beura L.K., Wijeyesinghe S., Thompson E.A., Macchietto M.G., Rosato P.C., Pierson M.J., Schenkel J.M., Mitchell J.S., Vezys V., Fife B.T. (2018). T Cells in Nonlymphoid Tissues Give Rise to Lymph-Node-Resident Memory T Cells. Immunity.

[177] McCully M.L., Kouzeli A., Moser B. (2018). Peripheral Tissue Chemokines: Homeostatic Control of Immune Surveillance T Cells. Trends Immunol..

[178] Zlotnik A., Yoshie O. (2012). The chemokine superfamily revisited. Immunity.

[179] Srivastava R., Hernandez-Ruiz M., Khan A.A., Fouladi M.A., Kim G.J., Ly V.T., Yamada T., Lam C., Sarain S.A.B., Boldbaatar U. (2018). CXCL17 Chemokine-Dependent Mobilization of CXCR8^+^CD8^+^ Effector Memory and Tissue-Resident Memory T Cells in the Vaginal Mucosa Is Associated with Protection against Genital Herpes. J. Immunol..

[180] Dhanushkodi N.R., Prakash S., Quadiri A., Zayou L., Srivastava R., Tran J., Dang V., Shaik A.M., Chilukurri A., Suzer B. (2023). Mucosal CCL28 Chemokine Improves Protection against Genital Herpes through Mobilization of Antiviral Effector Memory CCR10^+^CD44^+^ CD62L-CD8^+^ T Cells and Memory CCR10^+^B220^+^CD27^+^ B Cells into the Infected Vaginal Mucosa. J. Immunol..

[181] Chen Z., Haus J.M., Chen L., Wu S.C., Urao N., Koh T.J., Minshall R.D. (2020). CCL28-induced CCR10/eNOS interaction in angiogenesis and skin wound healing. FASEB J..

[182] Hernandez-Ruiz M., Zlotnik A. (2017). Mucosal Chemokines. J. Interferon Cytokine Res..

[183] Maravillas-Montero J.L., Burkhardt A.M., Hevezi P.A., Carnevale C.D., Smit M.J., Zlotnik A. (2015). Cutting edge: GPR35/CXCR8 is the receptor of the mucosal chemokine CXCL17. J. Immunol..

[184] Kim H.R., Hwang K.A., Park S.H., Kang I. (2008). IL-7 and IL-15: Biology and roles in T-Cell immunity in health and disease. Crit. Rev. Immunol..

[185] Nolz J.C., Richer M.J. (2020). Control of memory CD8^+^ T cell longevity and effector functions by IL-15. Mol. Immunol..

[186] Schenkel J.M., Fraser K.A., Casey K.A., Beura L.K., Pauken K.E., Vezys V., Masopust D. (2016). IL-15-Independent Maintenance of Tissue-Resident and Boosted Effector Memory CD8 T Cells. J. Immunol..

[187] Jabri B., Abadie V. (2015). IL-15 functions as a danger signal to regulate tissue-resident T cells and tissue destruction. Nat. Rev. Immunol..

[188] McGill J., Van Rooijen N., Legge K.L. (2010). IL-15 trans-presentation by pulmonary dendritic cells promotes effector CD8 T cell survival during influenza virus infection. J. Exp. Med..

[189] Jarjour N.N., Dalzell T.S., Maurice N.J., Wanhainen K.M., Peng C., DePauw T.A., Block K.E., Valente W.J., Ashby K.M., Masopust D. (2024). Collaboration between IL-7 and IL-15 enables adaptation of tissue-resident and circulating memory CD8^+^ T cells. bioRxiv.

[190] Shen C.H., Ge Q., Talay O., Eisen H.N., Garcia-Sastre A., Chen J. (2008). Loss of IL-7R and IL-15R expression is associated with disappearance of memory T cells in respiratory tract following influenza infection. J. Immunol..

[191] Roy S., Coulon P.G., Srivastava R., Vahed H., Kim G.J., Walia S.S., Yamada T., Fouladi M.A., Ly V.T., BenMohamed L. (2018). Blockade of LAG-3 Immune Checkpoint Combined With Therapeutic Vaccination Restore the Function of Tissue-Resident Anti-viral CD8^+^ T Cells and Protect Against Recurrent Ocular Herpes Simplex Infection and Disease. Front. Immunol..

[192] Srivastava R., Dervillez X., Khan A.A., Chentoufi A.A., Chilukuri S., Shukr N., Fazli Y., Ong N.N., Afifi R.E., Osorio N. (2016). The Herpes Simplex Virus Latency-Associated Transcript Gene Is Associated with a Broader Repertoire of Virus-Specific Exhausted CD8^+^ T Cells Retained within the Trigeminal Ganglia of Latently Infected HLA Transgenic Rabbits. J. Virol..

[193] Gola A., Silman D., Walters A.A., Sridhar S., Uderhardt S., Salman A.M., Halbroth B.R., Bellamy D., Bowyer G., Powlson J. (2018). Prime and target immunization protects against liver-stage malaria in mice. Sci. Transl. Med..

[194] Mohan T., Zhu W., Wang Y., Wang B.Z. (2018). Applications of chemokines as adjuvants for vaccine immunotherapy. Immunobiology.

[195] Elhoucine Elfatimi Y.L. (2025). Swayam Prakash, Lbachir BenMohamed, Artificial intelligence and machine learning in the development of vaccines and immunotherapeutics—Yesterday, today, and tomorrow. Front. Artif. Intell..

[196] Iwasaki A. (2016). Exploiting Mucosal Immunity for Antiviral Vaccines. Annu. Rev. Immunol..

[197] El Arab R.A., Alkhunaizi M., Alhashem Y.N., Al Khatib A., Bubsheet M., Hassanein S. (2025). Artificial intelligence in vaccine research and development: An umbrella review. Front. Immunol..

[198] Olawade D.B., Teke J., Fapohunda O., Weerasinghe K., Usman S.O., Ige A.O., Clement David-Olawade A. (2024). Leveraging artificial intelligence in vaccine development: A narrative review. J. Microbiol. Methods.

[199] Greiff V., Weber C.R., Palme J., Bodenhofer U., Miho E., Menzel U., Reddy S.T. (2017). Learning the High-Dimensional Immunogenomic Features That Predict Public and Private Antibody Repertoires. J. Immunol..

[200] Van Gassen S., Callebaut B., Van Helden M.J., Lambrecht B.N., Demeester P., Dhaene T., Saeys Y. (2015). FlowSOM: Using self-organizing maps for visualization and interpretation of cytometry data. Cytom. A.

[201] Cannoodt R., Saelens W., Saeys Y. (2016). Computational methods for trajectory inference from single-cell transcriptomics. Eur. J. Immunol..

[202] Sade-Feldman M., Yizhak K., Bjorgaard S.L., Ray J.P., de Boer C.G., Jenkins R.W., Lieb D.J., Chen J.H., Frederick D.T., Barzily-Rokni M. (2019). Defining T Cell States Associated with Response to Checkpoint Immunotherapy in Melanoma. Cell.

[203] Scott M., Lundberg S.-I.L. A unified approach to interpreting model predictions. Proceedings of the 31st International Conference on Neural Information Processing Systems.

[204] Vaswani A., Shazeer N., Parmar N., Uszkoreit J., Jones L., Gomez A.N., Polosukhin I. (2017). Attention Is All You Need. Sci. Res..

[205] Miho E., Yermanos A., Weber C.R., Berger C.T., Reddy S.T., Greiff V. (2018). Computational Strategies for Dissecting the High-Dimensional Complexity of Adaptive Immune Repertoires. Front. Immunol..

[206] Reynisson B., Alvarez B., Paul S., Peters B., Nielsen M. (2020). NetMHCpan-4.1 and NetMHCIIpan-4.0: Improved predictions of MHC antigen presentation by concurrent motif deconvolution and integration of MS MHC eluted ligand data. Nucleic Acids Res..

[207] Chen B., Khodadoust M.S., Olsson N., Wagar L.E., Fast E., Liu C.L., Muftuoglu Y., Sworder B.J., Diehn M., Levy R. (2019). Predicting HLA class II antigen presentation through integrated deep learning. Nat. Biotechnol..

[208] Chew T., Taylor K.E., Mossman K.L. (2009). Innate and adaptive immune responses to herpes simplex virus. Viruses.

[209] Coleman J.L., Shukla D. (2013). Recent advances in vaccine development for herpes simplex virus types I and II. Hum. Vaccin. Immunother..

[210] Malik S., Sah R., Ahsan O., Muhammad K., Waheed Y. (2023). Insights into the Novel Therapeutics and Vaccines against Herpes Simplex Virus. Vaccines.

[211] Awasthi S., Friedman H.M. (2014). Status of prophylactic and therapeutic genital herpes vaccines. Curr. Opin. Virol..

[212] Greenbaum J.A., Kotturi M.F., Kim Y., Oseroff C., Vaughan K., Salimi N., Vita R., Ponomarenko J., Scheuermann R.H., Sette A. (2009). Pre-existing immunity against swine-origin H1N1 influenza viruses in the general human population. Proc. Natl. Acad. Sci. USA.

[213] Feng Y., Jiang H., Qiu M., Liu L., Zou S., Li Y., Guo Q., Han N., Sun Y., Wang K. (2021). Multi-Epitope Vaccine Design Using an Immunoinformatic Approach for SARS-CoV-2. Pathogens.

[214] Mamun T.I., Ali M.A., Hosen M.N., Rahman J., Islam M.A., Akib M.G., Zaman K., Rahman M.M., Hossain F.M.A., Ibenmoussa S. (2024). Designing a multi-epitope vaccine candidate against human rhinovirus C utilizing immunoinformatics approach. Front. Immunol..

[215] Nagpal G., Usmani S.S., Dhanda S.K., Kaur H., Singh S., Sharma M., Raghava G.P. (2017). Computer-aided designing of immunosuppressive peptides based on IL-10 inducing potential. Sci. Rep..

[216] Ahmed S.F., Quadeer A.A., McKay M.R. (2020). Preliminary Identification of Potential Vaccine Targets for the COVID-19 Coronavirus (SARS-CoV-2) Based on SARS-CoV Immunological Studies. Viruses.

[217] Ariotti S., Beltman J.B., Chodaczek G., Hoekstra M.E., van Beek A.E., Gomez-Eerland R., Ritsma L., van Rheenen J., Maree A.F., Zal T. (2012). Tissue-resident memory CD8^+^ T cells continuously patrol skin epithelia to quickly recognize local antigen. Proc. Natl. Acad. Sci. USA.

[218] Iijima N., Iwasaki A. (2014). T cell memory. A local macrophage chemokine network sustains protective tissue-resident memory CD4 T cells. Science.

[219] Milner J.J., Toma C., Yu B., Zhang K., Omilusik K., Phan A.T., Wang D., Getzler A.J., Nguyen T., Crotty S. (2018). Erratum: Runx3 programs CD8^+^ T cell residency in non-lymphoid tissues and tumours. Nature.

[220] Xu L., Ye L., Huang Q. (2025). Tissue-Resident Memory CD8^+^ T Cells: Differentiation, Phenotypic Heterogeneity, Biological Function, Disease, and Therapy. MedComm.

[221] Schleiss M.R. (2013). Developing a Vaccine against Congenital Cytomegalovirus (CMV) Infection: What Have We Learned from Animal Models? Where Should We Go Next?. Future Virol..

[222] Sette A., Rappuoli R. (2010). Reverse vaccinology: Developing vaccines in the era of genomics. Immunity.

[223] Soleymani F., Paquet E., Viktor H., Michalowski W., Spinello D. (2022). Protein-protein interaction prediction with deep learning: A comprehensive review. Comput. Struct. Biotechnol. J..

[224] Gebhardt T., Wakim L.M., Eidsmo L., Reading P.C., Heath W.R., Carbone F.R. (2009). Memory T cells in nonlymphoid tissue that provide enhanced local immunity during infection with herpes simplex virus. Nat. Immunol..

[225] Paul S., Weiskopf D., Angelo M.A., Sidney J., Peters B., Sette A. (2013). HLA class I alleles are associated with peptide-binding repertoires of different size, affinity, and immunogenicity. J. Immunol..

[226] Walker L.J., Sewell A.K., Klenerman P. (2010). T cell sensitivity and the outcome of viral infection. Clin. Exp. Immunol..

